# Genetic Responses Induced in Olive Roots upon Colonization by the Biocontrol Endophytic Bacterium *Pseudomonas fluorescens* PICF7

**DOI:** 10.1371/journal.pone.0048646

**Published:** 2012-11-07

**Authors:** Elisabetta Schilirò, Massimo Ferrara, Franco Nigro, Jesús Mercado-Blanco

**Affiliations:** 1 Departamento de Protección de Cultivos, Instituto de Agricultura Sostenible, Consejo Superior de Investigaciones Científicas (CSIC), Córdoba, Spain; 2 Dipartimento di Biologia e Chimica Agro-forestale ed Ambientale, Università degli Studi di Bari “Aldo Moro”, Bari, Italy; Universidad Pública de Navarra, Spain

## Abstract

Knowledge on the genetic basis underlying interactions between beneficial bacteria and woody plants is still very limited, and totally absent in the case of olive. We aimed to elucidate genetic responses taking place during the colonization of olive roots by the native endophyte *Pseudomonas fluorescens* PICF7, an effective biocontrol agent against Verticillium wilt of olive. Roots of olive plants grown under non-gnotobiotic conditions were collected at different time points after PICF7 inoculation. A Suppression Subtractive Hybridization cDNA library enriched in induced genes was generated. Quantitative real time PCR (qRT-PCR) analysis validated the induction of selected olive genes. Computational analysis of 445 olive ESTs showed that plant defence and response to different stresses represented nearly 45% of genes induced in PICF7-colonized olive roots. Moreover, quantitative real-time PCR (qRT-PCR) analysis confirmed induction of lipoxygenase, phenylpropanoid, terpenoids and plant hormones biosynthesis transcripts. Different classes of transcription factors (i.e., bHLH, WRKYs, GRAS1) were also induced. This work highlights for the first time the ability of an endophytic *Pseudomonas* spp. strain to mount a wide array of defence responses in an economically-relevant woody crop such as olive, helping to explain its biocontrol activity.

## Introduction

Olive (*Olea europaea* L.) is an evergreen, long-living woody species cultivated worldwide in Mediterranean-type climatic regions. Cultivation of olive trees for millennia has modulated the landscape of large geographical areas, creating a very specific agroecosystem and sustaining an important agroindustry (olive oil and table olive production). It has an outstanding social and economical relevance in many countries, particularly within the Mediterranean basin [1].

Verticillium wilt, caused by the soil-borne fungus *Verticillium dahliae* Kleb., is currently considered one of the most serious biotic constraints for olive cultivation. The disease is very difficult to control and it can only be effectively confronted by implementing an integrated disease management strategy [2]. An interesting measure to control Verticillium wilt of olive (VWO), which can be used in combination with others mainly at the nursery production stage, is the use of biological control agents (BCAs). However, few examples on the effective use of BCAs against VWO are available [2], [3].

An often dominant bacterial group found in the rhizosphere of diverse *Verticillium*-susceptible crops, including olive, is the genus *Pseudomonas* [4], [5], which comprises diverse well-studied plant-growth promoting and biocontrol strains [6]. *Pseudomonas fluorescens* PICF7 is a natural colonizer of olive roots and an effective BCA against VWO [7]. Its ability to endophytically colonize root tissues of different olive cultivars has been demonstrated under non-gnotobiotic [8] and gnotobiotic [9] experimental conditions. Effective suppression of the disease in *V. dahliae* artificially-inoculated olive plants seems to require the early establishment of strain PICF7 at both the surface and the interior of olive root tissues, prior to root colonization by the pathogen [10]. The use of beneficial endophytic bacteria is of particular interest in plant health management. This is due to their ability to internally colonize plant tissues thus being adapted to the ecological niche where biocontrol mechanism(s) may be deployed, potentially triggering a long-term plant protection status [11], [12].

Among the mechanisms by which beneficial soil bacteria contribute to plant growth and health, the induction of plant defence responses against soil-borne pathogens represents a very attractive tool to be harnessed in modern sustainable agricultural practices [13], [14]. Plant-mediated defence response by plant growth-promoting rhizobacteria (PGPR) [15] can be exerted through different mechanisms such as root architecture modification, production of phytohormones and antibiotic compounds, and triggering of specific defence signalling pathway known as induced systemic resistance (ISR) [16], [17], [18]. The characteristic responses of systemic defence mechanisms activated in plants whose roots are colonized by PGPR have been early described [19] and include the induction of enzymatic activities related to peroxidases (PO), polyphenoloxidases (PPO) or phenylalanine ammonia-lyase (PAL) synthesis as well as increased transcription levels of genes coding for pathogen-related (PR) proteins such as β-1,3-glucanases and chitinases. The ISR response elicited by non-pathogenic rhizobacteria is phenotypically similar to systemic acquired resistance (SAR), an induced plant defence response associated with local and systemic increases of endogenously-synthesized salicylic acid (SA) and coupled to a coordinated expression of PR genes. Both beneficial and pathogenic bacteria can promote cell wall reinforcement, and production of phytoalexins and PR proteins, consistent with the fact that SAR and ISR represent an enhanced basal plant resistance [20].

Mechanisms underlying the interaction between beneficial rhizobacteria and woody plants remain largely unknown, particularly at the transcriptional level. In the case of olive, information concerning gene expression during colonization by beneficial bacteria is totally absent. In this study, we aimed to elucidate the genetic processes taking place during the colonization of olive roots by the native, beneficial endophyte *P. fluorescens* PICF7 under non-gnotobiotic conditions. A Suppression Subtractive Hybridization (SSH) cDNA library from roots of olive plants (cv. Arbequina) inoculated with strain PICF7 was generated, enabling the identification of up-regulated olive genes. Results suggest that this beneficial, endophytic *Pseudomonas* strain is able to trigger a wide array of defence responses in olive roots which can explain its biocontrol effectiveness against VWO.

## Materials and Methods

### Plant Material, *Pseudomonas Fluorescens* PICF7 Root Treatment, and mRNA Purification

Three-month-old, nursery-produced potted olive plants of the Verticillium wilt-susceptible cv. Arbequina [21] were acclimated for three weeks in a growth chamber prior to bacterial treatment. *Pseudomonas fluorescens* strain PICF7 [7] inoculum was prepared from the bacterial biomass grown on LB agar plates at 25°C for 48 h as previously described [8]. Olive plants were carefully uprooted from the original substrate, their roots gently washed under tap water and dipped for 15 min in a 1·10^8^ cells ml^−1^ strain PICF7 suspension (inoculated samples) or in 10 mM MgSO_4_·7 H_2_O (control samples). Plants were then individually transplanted into polypropylene pots filled with an autoclaved sandy substrate prepared *ad hoc* [8], and incubated in a growth chamber adjusted to 23±1°C, 60–90% relative humidity and a 14-h photoperiod of fluorescent light at at 360 µE m^−2^ for 21 days.

In order to collect a wide range of differentially-expressed (induced) genes, the entire root systems were sampled at different times after treatments. Thus, roots were harvested at 0 h, 5 h, 10 h, 24 h and 2, 3, 4, 7, 10, 12, 15, 18 and 21 days (two plants/time point) after bacterization or 10 mM MgSO_4_·7H_2_O treatments. A total of 52 plants were used. Root tissue samples were immediately frozen in liquid nitrogen and kept at −80°C until RNA extraction. Total RNA was isolated separately from inoculated and control samples for each time point according to Asif and coworkers [22]. Contaminating genomic DNA was removed by DNaseI treatment (Roche Applied Science, Mannheim, Germany) according to the manufacturer’s indications. Previous to mRNA purification, all RNA samples corresponding to each treatment (PICF7-inoculated and control plants) were pooled separately to obtain two independent RNA pools. Poly A+ mRNA was finally purified from about 400 µg of total RNA from each pool using the Dynabeads® mRNA Purification Kit (Dynal Biotech, Oslo, Norway) according to the manufacturer’s instructions. The mRNA purity and quality was checked by both agarose gel electrophoresis and spectrophotometry using a ND-1000 Spectrophotometer (NanoDrop Technologies, Wilmington, DE, USA).

### Generation of a Suppression Substractive Hybridization (SSH) cDNA Library, Cloning and Sequencing

To clone and identify genes induced by *P. fluorescens* PICF7 in olive root tissues, a cDNA library was generated by using the Suppression Subtractive Hybridization (SSH) methodology [23]. This approach enables the enrichment and cloning of less abundant transcripts through amplification and normalization of subtracted cDNAs. The SSH library was generated using the PCR-Select™ cDNA Subtraction Kit (BD Biosciences, Palo Alto, CA, USA) following the manufacturer’s instructions. Briefly, cDNAs were separately synthesized from 2 µg of mRNA of each PICF7-inoculated (tester) and control (driver) plants, digested with *RsaI* and ligated to adaptors 1 and 2R. Two rounds of hybridization and PCR amplification were then performed to enrich differentially-expressed sequences. PCR amplification was carried out using the Advantage® 2 PCR Kit (BD Biosciences, Palo Alto, CA, USA) in 50 µL final volume. Amplification conditions consisted of 5 min of denaturation at 94°C, followed by 33 cycles of 30 s at 94°C, 45 s at 55°C and 1 min at 72°C, with a final extension step of 10 min at 72°C. The efficiency of subtraction was checked by amplifying a 308-bp fragment defined by the primer pair Act1-fw: 5′-GCTTGCTTATGTTGCTCTCGAC-3′/Act1-rv: 5-TGATTTCCTTGCTCATACGGTC-3′) of the constitutively expressed (housekeeping) β-actin gene from olive (Acc. No. AF545569), whose expression was checked not to be influenced by strain PICF7 inoculation (data not shown).

Enriched subtracted cDNAs generated after SSH were ligated in the pGEM-T Easy Vector (Promega, Madison, WI, USA) and cloned into *Escherichia coli* CH3-Blue competent cells (Bioline, London, UK) according to manufacturer’s procedures. Positive transformants based on white/blue colour selection were grown in 96-well microtiter plates in LB medium with 100 mg L^−1^ ampicillin at 37°C for 22 hours. One thousand bacterial clones of the resulting SSH library were eventually isolated and sequenced by using the forward T7 universal primer. DNA sequencing was performed at Sistemas Genómicos S.L., (Valencia, Spain).

### Bioinformatics Analysis of Expressed Sequences Tags

The complete set of Expressed Sequence Tags (ESTs) was trimmed from vector and adaptors sequences contamination by mass alignment using the ‘CLC Main Workbench 6′ (CLC bio, Aarhus, Denmark) software. Low quality and short (<100 bp) sequences were excluded from the analysis. After this ‘sequence cleaning’ step, the ‘CLC Main Workbench 6′ software was used to assemble the EST data set in order to find contiguous sequences and redundancy. Computational annotation of ESTs from the olive-PICF7 interaction was performed using the public software ‘Blast2GO version 2.4.5’ [24] available at http://www.blast2go.com/b2ghome. Homologies were checked in the non-redundant GenBank protein database by running the Blastx algorithm [25] with the E-value set to 1.0e^−3^ and the High-scoring Segment Pairs (HSP) length cutoff fixed to 33.

Similarly, the ‘Blast2GO software v.2.4.5’ was used to obtain Gene Ontology (GO) information from retrieved database matches. Annotation of all sequences was performed by using default parameters. InterPro Scan [26] analysis was applied to find functional motifs and related GO terms by using the specific tool implemented in the Blast2GO software with the default parameters. Finally, the ‘Augment Annotation by ANNEX’ function was used to improve the annotation profiles information. Both the GOslim ‘goslim_generic.obo’ (used to achieve all GO terms) and the GOslim ‘goslim_plant.obo’ (used to achieve specific plant GO terms by means of a plant-specific reduced version of the GO available at http://www.arabidopsis.org/) were run. Enzyme mapping of annotated sequences was retrieved by direct GO to Enzyme annotation and used to query the Kyoto Encyclopaedia of Genes and Genomes (KEGG - http://www.genome.jp/kegg/) to define the main metabolic pathways involved.

### Data Validation and Expression Profile Analysis by Quantitative Real-Time PCR

Quantitative Real-Time PCR (qRT-PCR) experiments were carried out to validate the induction of some of the ESTs identified in the previous bioinformatics analysis. Among the whole dataset of non-redundant sequences, nine transcripts belonging to key biosynthetic and metabolic pathways were selected according to the length (>100 bp) and E-value (≤1.0e^−5^). Specific primer pairs for each sequence were designed using the ‘Primer3Plus’ software [27] and tested for their specificity at 55°C by conventional PCR. To determine the range of concentrations at which target cDNA and cycle thresholds (Ct) values were linearly correlated and to estimate PCR efficiencies, specific qRT-PCR assays were conducted using cDNAs synthesized from 10-fold serially diluted (1 µg, 100 ng, 10 ng, 1 ng, 100 pg) RNA samples. For each selected gene, relative standard curves were constructed using reverse transcribed cDNA from serial dilutions of total RNA of a “Reference Sample” of the same pooled total RNA used for the subtraction experiment. Ct values and the logarithm of cDNA concentrations were linearly correlated for each of the examined genes. cDNA was synthesized by reverse transcription of 100 ng of total RNA using iScript cDNA Synthesis Kit (BioRad Hercules, CA, USA) following the manufacturer’s instructions. qRT-PCR analyses were finally performed using a thermal cycler iQ5 Real-Time PCR System (BioRad) equipped with a 96 well plates. For all transcripts, reactions were repeated at least two times (independent qRT-PCR experiments) and each of these assays always included three biological replicas per transcript and per plate.

All qRT-PCR experiments were carried out in a final volume of 20 µL containing 2 µL of cDNA, 0.5 µM of each primer, and 10 µL of 2 × iQ™ SYBR® Green Supermix (BioRad) according to the manufacturer’s procedures. The following thermal cycling profile was used for all PCR reactions: 94°C for 5 min, 50 cycles of 94°C for 30 s, 55°C for 30 s and 72°C for 40 s. Linear equations, correlation coefficients (R^2^) and reaction efficiencies were calculated in all cases. Melting curves of qRT-PCR products were assessed from 55°C to 95°C to confirm the amplification of single PCR bands. Reaction conditions were as follows: initial denaturation for 5 min at 95°C, cooling to 55°C and melting from 55°C to 95°C with a 0.5°C transition rate every 10 sec.

Data resulting from qRT-PCR were normalized to the *O. europaea* β-actin gene used as housekeeping gene and amplified in the same conditions. Relative expression (RE) levels were calculated according to Livak and Schmittgen [28]. The average of each expression gene fold change was categorized as follows: “low” ≥ −1.0 to ≤1.0; “medium” ≥ −2.0 to < −1.0 or >1.0 to ≤2.0; “high” < −2.0 or >2.0 [29].

### Accession Numbers

ESTs reported in this study have been deposited in the dbESTs database of the National Center for Biotechnology Information (NCBI) under GenBank accession numbers JK755245 (dbEST_Id 76225223) - JK756148 (dbEST_Id 76226126).

## Results

### Identification of Olive Genes Induced during the Colonization Process of Olive Roots by *Pseudomonas Fluorescens* PICF7

A forward subtracted cDNA library enriched in up-regulated olive transcripts and represented by 904 high-quality EST sequences was generated. After assembling contiguous and overlapping clones, 196 distinct contigs (average length 438 bp) and 249 singlets (average length 396 bp) were identified, enabling the characterization of 445 independent EST sequences differentially expressed (induced) during PICF7-olive interaction. The library redundancy (number of sequences assembled into contigs/total number of sequences analyzed) was relative low and represented 7.2% of the entire ESTs set.

Querying (Blastx) the non-redundant NCBI database allowed the attribution of homologous hits for 94% of the ESTs. 195 ESTs (43.8% of the whole ESTs set) represented coding sequences present in genomes of woody plants such as grape vine (*Vitis vinifera* L., 85 hits), castor bean (*Ricinus communis* L., 53 hits), western balsam poplar (*Populus trichocarpa*, 46 hits) and olive (9 hits) (Table 1). E-values ranged from 1.6e^−4^ to 2.02e^−112^. Remarkably, only 2.5% of the 445 unique EST sequences showed significant identity to sequences found in the databases for olive (6,067 entries in the NCBI dbEST [http://www.ncbi.nlm.nih.gov/dbEST/], last search on March 2012), stressing the current lack of genetic information for this relevant woody crop. Interestingly, none of the ESTs homologous to the olive transcripts identified in this work has been previously reported as involved in any olive-biotic interaction. Finally, Blastx analysis classified 85 sequences (19% of the unique EST data set here reported) as unknown. These ESTs represent interesting candidates for novel genes putatively involved in the interaction of a beneficial, endophytic bacterium with olive roots. Analysis of the 445 olive ESTs showed that events associated with plant defence and response to stresses represented more than 40% of the genes induced in olive roots after PICF7 inoculation. Relevant ESTs identified as induced by *P. fluorescens* PICF7 during olive roots colonization are shown in Table S1. Contigs identified in this study along with their corresponding contiguous/overlapping ESTs are shown in Table S2. Transcripts coding for unknown function were not included in these tables.

**Table 1:** *Pseudomonas fluorescens* PICF7-induced EST sequences identified after Blastx analysis as homologous to olive (*Olea europaea*) genes previously included in databases.

EST Sequence Name	Putative protein function	Accession Number	E-Value
ARBRI-C23**^1^**	mip pip subfamily	ABB13429	1,52E-14
ARBRI-C30	Drought-induced protein sdi-6-like	ABS72020	4,42E-46
ARBRI-C67**^2^**	Cardenolide 16-o-glucohydrolase	AAL93619	3,06E-23
ARBRI-C89**^2^**	Glycoside hydrolase family 1 protein	AAL93619	4,83E-45
ARBRI-C93	Cold-induced glucosyl transferase	ACW82415	1,13E-25
ARBRI-C134**^1^**	Plasma membrane intrinsic protein	ABB13429	8,15E-77
ARBRI-2_T7_F05	Lipoxygenase	ACG56281.1	4,81E-70
ARBRI-2_T7_H05**^3^**	β-glucanase	CAH17549	1,08E-36
ARBRI-4_T7_D01	Hexose transporter	ABJ98314.1	2,05E-80
ARBRI-4_T7_H08**^3^**	β-glucanase	CAH17549	2,80E-37
ARBRI-9_T7_E04	Apyrase-like protein	ABS72007.1	5.30E-67
ARBRI-10_T7_E01	Plasma membrane intrinsic protein	ABB13430.2	1.59E-48

Sequence names, putative protein functions, accession numbers and E-values are indicated.

ESTs ARBRI-C23 and ARBRI-C134^(1)^, ARBRI-C67 and ARBRI-C89^(2)^, and ARBRI-2_T7_H05 and ARBRI-4_T7_H08^(3)^ showed similarity to the same olive accessions in the databases but at different sequence regions. The different protein regions to which they showed similarity were identified by BLASTx algorithm under different names, except for the olive β-glucanase (Accession Number CAH17549).

### Computational Analysis of Genes Induced in Olive Roots by *P. Fluorescens* PICF7

**Figure 1:**
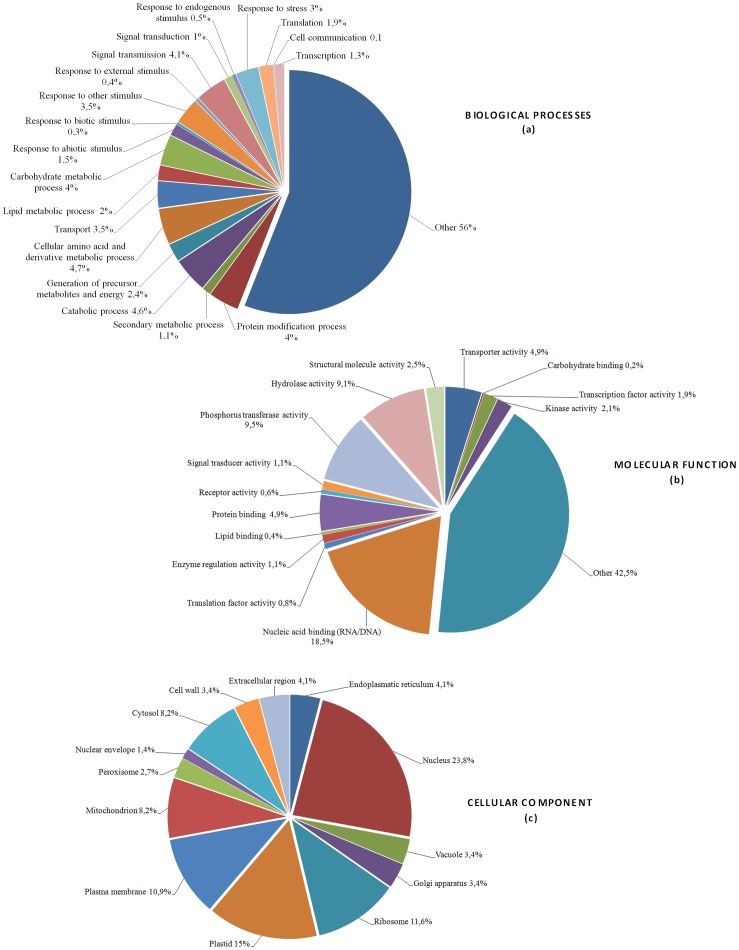
Gene Ontology (GO) terms distribution of 341 unigenes induced in olive (*Olea europaea*) roots colonized by *Pseudomonas fluorescens* PICF7. Expressed sequences tags (ESTs) were categorized using the ‘Blast2GO’ software according to the terms of the three main GO vocabularies: ‘biological processes’ (a), ‘molecular functions’ (b) and ‘cellular components’ (c). The category ‘Other’ in the main GO vocabulary term ‘biological processes’ clusters 56% of the transcripts analyzed and refers to very low represented categories related to development, homeostasis, photosynthesis and cellular differentiation. In the main GO vocabulary term ‘molecular functions’, the category ‘Other’ clusters 42.5% of ESTs with similarity to proteins with putative or unknown function (see text for details).

Analysis of the EST set by the Blast2GO software allowed the annotation of expressed sequences according to the terms of the three main GO vocabularies, *i*.*e*. ‘biological process’, ‘molecular function’ and ‘cellular component’. It is worth mentioning that only 76.6% of the sequences were mapped by the GO annotation database. Thus, 104 ESTs (85 unknown and 19 predicted sequences corresponding to the remaining 23.4%) were not included in this functional classification because they were automatically discarded by the program before running the analysis. Since some translated sequences were identified by different GO terms, they were classified according to the best hits retrieved from the BLAST homology search. ESTs distribution for ‘biological processes’, ‘molecular function’ and ‘cellular component’ main categories are shown in Figure 1.

Regarding to biological processes, 56% (190 sequences) of the transcripts corresponded to very low represented GO terms categories (i.e., cell cycle, flower and reproductive structures development, cellular homeostasis, photosynthesis, pollination, cell differentiation, embryonic and post-embryonic development), and were grouped as ‘other’ in our analysis (Fig. 1A). Concerning to the remaining EST sequences induced by *P. fluorescens* PICF7 colonization (44%, 151 sequences) more than 10% of them were assigned to categories such as signal transmission (4%) and transduction (1%), response to stress (3%), transcription (1.3%) and translation (1.9%). GO terms included lipoxygenase (ARBRI-2_T7_F05), catalases (ARBRI-C50; ARBRI-2_T7_C02; ARBRI-2_T7_C12; ARBRI-10_T7_B01) and proteins involved in phenylpropanoid pathways (e.g. caffeoyl-O-methyltransferase [ARBRI-1_T7_B01], PAL [ARBRI-C73], cinnamyl-alcohol dehydrogenase [ARBRI-1-_T7_F06]), or terpenoids and hormones biosynthesis such as serine/threonine kinases (ARBRI-10_T7_E11; ARBRI-10_T7_H01), acetone-cyanohydrin lyase (ARBRI-C140) and malate dehydrogenases (ARBRI-C51; ARBRI-10_T7_F09). In addition, we identified as induced by *P. fluorescens* PICF7 colonization transcripts belonging to different classes of PR proteins such as β-1,3 glucanases (PR-2) (ARBRI-2_T7_H05; ARBRI-C123), class IV endochitinases belonging to PR-4 family (ARBRI-5_T7_A04) and to PR-3 family (ARBRI-10_T7_A06), peroxidases (PR-9) (ARBRI-10_T7_B05; ARBRI-5_T7_F05; ARBRI-5_T7_A09; ARBRI-3_T7_D04) and Bet_v1-like proteins (PR-10) (ARBRI-1_T7_E07; ARBRI-C27A), which have been previously reported as part of the plant ISR response triggered by PGPRs [18].

Interestingly, the most represented category (6.2% of the genes up-regulated after PICF7 inoculation) within biological processes was constituted by responses to different kind of stimulus (endogenous [0.5%], external [0.4%], biotic [0.3%], abiotic [1.5%] and ‘other stimulus’ [3.5%]) (Fig. 1A). To these stimulus-response categories were assigned aldehyde dehydrogenases (ARBRI-C14;ARBRI-C21; ARBRI-4_T7_C05; ARBRI-4_T7_G12;ARBRI-10_T7_F12), an aldo keto reductase (ARBRI-10_T7_F01), a choline/ethanolamine kinase (ARBRI-10_T7_F05), glutathione-S-transferases (ARBRI-C18; ARBRI-3_T7_D07; ARBRI-6_T7_B12), a β-amylase (ARBRI-C92), and 14 Kda proline-rich-proteins (ARBRI-7_T7_C08; ARBRI-10_T7_C06). Finally, seven of the transcripts assigned to this category encoded for proteins with unknown function.

Although numerically less represented, translation (1.9%) and transcription (1.3%) categories (Fig. 1A) included signal transcription factors known to be involved in plant response to pathogens such as binding factors responsive to jasmonic acid (bHLH) (ARBRI-C74), auxin (TF2) (ARBRI-C110), salicylic acid (WRKYs) (ARBRI-C83; ARBRI-C86) and a myb-like DNA binding domain factor (ARBRI-C71). Up to 3.5% of the ESTs identified as PICF7-induced were related to transport processes. GO terms assigned to this category included metal and ion transport (ZIP family iron transporters, metal ion binding proteins) (ARBRI-C27C; ARBRI-C153), carbohydrate carriers (ARBRI-1_T7_E05; ARBRI-2_T7_C01; ARBRI-4_T7_D01) and plasma membrane intrinsic proteins (ARBRI-10_T7_E01; ARBRI-C23; ARBRI-6_T7_D05). Approximately 15% of the PICF7-induced ESTs corresponded to genes involved in different classes of primary and secondary metabolism for carbohydrates, amino acids and lipids (representing 11.8% of the EST sequences included in the computational analysis), catabolic processes (4.6%) and protein modification (4%) (Fig. 1A). Transcripts involved in precursor metabolites and energy generation represented 2.4% of the genes expressed upon *P. fluorescens* PICF7 inoculation. Finally, cell communication was also represented (0.1%) by a purine permease (ARBRI-C161), a catalase (ARBRI-2_T7_C02) and a LEA (late embryogenesis abundant) 3 like protein (ARBRI-10_T7_D02).

Even though more than 42% of the transcripts could not be assigned to any specific function (classified as ‘other’) when the main GO vocabulary term ‘molecular function’ was searched, 14 different categories could be identified (Fig. 1B). The analysis revealed a high proportion of signalling events induced by *P. fluorescens* PICF7 root colonization. Thus, the category nucleic acid binding was the most represented (18.5%), followed by phosphorus transferase (9.5%), protein binding (4.9%), kinase (2.1%), transcription factor (1.9%), signal transducer (1.1%) and receptor (0.6%) activities. Finally, additional functions elicited by PICF7 in olive roots included hydrolases (9.1%) and transport (4.9%) activities, whereas other molecular functions were represented at lower extent (Fig. 1B).

With respect to the main GO vocabulary term ‘cellular component’, the most represented compartment was the nucleus (23.8%) (Fig. 1C), in agreement with the biological processes and molecular functions mentioned above, suggesting an important activity at the transcriptional level. On the other hand, 3.4% of the entries corresponded to proteins associated to plant cell wall such as α-1,4-glucan-protein synthase (required for cell wall polysaccharides synthesis) (ARBRI-C183) and glycosyl hydrolases (ARBRI-123; ARBRI-2_T7_H05). This is in agreement with earlier reports linking induced defence responses by endophytic PGPR to structural cell wall modifications [30].

**Figure 2:**
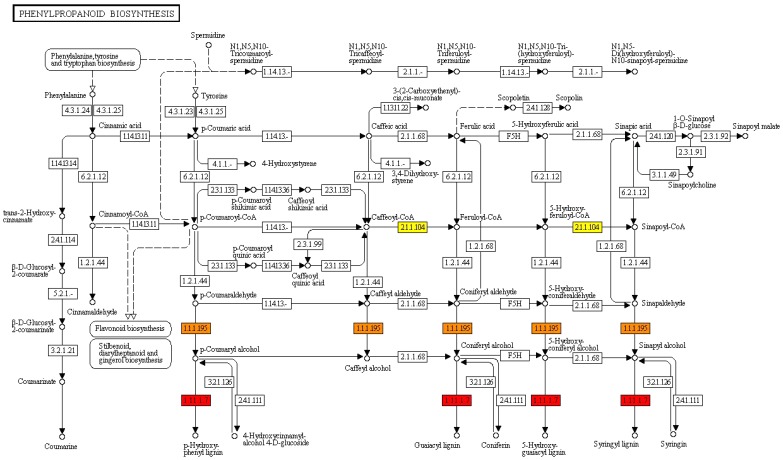
Kyoto Encyclopaedia of Genes and Genomes (KEGG) for the phenylpropanoid biosynthesis pathway. Several genes involved in this pathway have been found to be induced in olive roots upon *Pseudomonas fluorescens* PICF7 colonization. The caffeoyl-CoA O-methyltransferase (2.1.1.104) (yellow rectangles), the cinnamyl-alcohol deydrogenase (1.1.1.95) (orange rectangles) and the peroxidase (1.11.1.7) (red rectangles) enzymes are mapped (see text for details and also Table 2).

Finally, the KEGG database was queried to associate sequences encoding enzymes and the deduced gene products to specific metabolic and/or biosynthetic pathways activated during PICF7 olive root colonization. KEGG maps analysis pointed out a transcriptional up-regulation of the majority of enzymes related to phenylpropanoids and plant hormones biosynthesis (Table 2 and Fig. 2).

**Table 2:** ESTs related to phenylpropanoids and plant hormones biosynthesis induced in olive roots (*Olea europaea*) by *Pseudomonas fluorescens* PICF7.

PHENYLPROPANOID AND HORMONES BIOSYNTHESIS			
Enzyme	E.C. number	Number of EST clones	GO categories for biological process
Chorismate synthase	4.2.3.5	5	Biosynthetic, amino acid and secondary metabolic processes
Malate dehydrogenase	1.1.1.37	2	Response to stress. Response to abiotic and biotic stimulus. Biosynthetic and amino acid metabolic processes
Peroxidase	1.11.1.7	4	Response to stress. Metabolic and catabolic processes
Caffeoyl-CoA O-methyltransferase	2.1.1.104	1	Biosynthetic, amino acid and secondary metabolic processes
Phosphoglycerate kinase	2.7.2.3	4	Biosynthetic and catabolic processes
Cinnamyl-alcohol dehydrogenase	1.1.1.195	1	Biosynthetic, amino acid and secondary metabolic processes
Pyruvate dehydrogenase (acetyl-transferring)	1.2.4.1	14	Metabolic and catabolic processes. Generation of energy
Glyceraldehyde-3-phosphate dehydrogenase (phosphorylating)	1.2.1.12	3	Metabolic and catabolic processes
Phosphoglycerate mutase	5.4.2.1	1	Response to stress. Metabolic and catabolic processes
Aspartate transaminase	2.6.1.1	1	Response to stress and to abiotic stimulus. Biosynthetic and amino acid metabolic processes
Lipoxygenase	1.13.11.12	1	Biosynthetic and lipid metabolic processes
4-hydroxy-3-methylbut-2-enyl diphosphate reductase	1.17.1.2	1	Catalytic activity
3-deoxy-7-phosphoheptulonate synthase	2.5.1.54	3	Biosynthesis

Enzyme name, EC number, the number of EST clones identified and the Gene Ontology (GO) categories for ‘biological processes’ are indicated according to the Kyoto Encyclopaedia of Genes and Genomes (KEGG) database. See also Figure 2 to map peroxidase (1.11.1.7), caffeoyl-CoA O-methyltransferase (2.1.1.104), and cinnamyl-alcohol dehydrogenase (1.1.1.195).

### Validation of PICF7-induced Defence Response Olive Genes by Quantitative Real-Time PCR

To investigate whether colonization by strain PICF7 triggers defence responses in olive roots, a group of genes identified as induced and putatively involved in the synthesis of PAL, acetone cyanohydrin lyase, lipoxygenase, caffeoyl-O-methyltransferase, and malate dehydrogenase, as well as the transcription factors WRKY 5, basic helix-loop-helix (bHLH), auxin responsive factor 2 (ARF2) and GRAS1 were selected for validation by qRT-PCR analysis.

qRT-PCR analyses validated the results from the generated SSH cDNA library for these selected genes. Linear equations, correlation coefficients (R^2^) and PCR efficiencies for each case are shown in Table 3. Data showed a general increase in the transcription levels of the analyzed genes and the relative fold change were assigned to three categories of up-regulation: high (>+2), medium (>+1.0 to ≤+2.0) and low (≥ −1.0 to ≤+1.0), according to Kim and coworkers [29]. In particular, the RE level of the acetone cyanohydrin lyase transcript resulted up-regulated at a high-level, with an estimated fold change of +2.85 log units compared with the control (non-bacterized) sample (Table 3). The increase of lipoxygenase gene expression level was also confirmed (+1.47) as well as the increased level for malate dehydrogenase (+1.2), caffeoyl-O-methyltransferase (+1.07) and PAL (+0.75) transcripts. Finally, up-regulation of the WRKY 5, bHLH, ARF2 and GRAS1 transcription factors was also corroborated by qRT-PCR analysis, with high up regulation levels for WRKY 5 and GRAS1 (+1.94 and +1.85, respectively). On the other hand, the relative fold change of the bHLH and the ARF2 factors was increased at medium (+1.04) or low (+0.7) level, respectively (Table 3).

**Table 3:** Relative expression (RE) of selected transcripts induced in olive roots upon *Pseudomonas fluorescens* PICF7 colonization.

Clone ID	Putative gene	Process	Primer pair	Amplicon length	Linear equation	R^2^	PCR efficiency	qRT-PCR (log_2_ fold-change)
ARBRI-C73	Phenylalanineammonia-lyase	Phenylpropanoids biosynthesis	Fw: AGGATTTGCTTCGAGTGGTT Rv: GGCAATTCTTGCACTCTCAA	230 bp	y = −3.34x+28.115	0.99	99%	+0.75
ARBRI-C140	Acetone cyanohydrinlyase	Salicylic acid-binding protein 2	Fw: GAAAGAGATGGAAGCGGAAA Rv: ACACAGGGAAATGCATCAAA	246 bp	y = −3.40x+24.665	0.99	96.6%	+2.85
ARBRI-1_T7_B01	Caffeoyl-O-methyltransferase	Phenylpropanoids biosynthesis	Fw: ACCAGAGGCCATGAAAGAAC Rv : ATTGCCAAAATCTTCCCATC	204 bp	y = −3.46x+15.528	0.99	94.5%	+1.07
ARBRI-2_T7_F05	Lipoxygenase	Jasmonic acid biosynthesis	Fw: TATCTCCCGGTTCGTCCTAC Rv: CCTCAACTTCGTCCCAAATC	264 bp	y = −3.12x+30.808	0.99	108.8%	+1.47
ARBRI-C51	Malatedehydrogenase	Biosynthesis of plant hormones	Fw: AGAATTGGATTTGGGTGAGC Rv: TTAATGGGGTCCCAGATGTT	242 bp	y = −3.52x +22.21	0.98	92.3%	+1.2
ARBRI-C83	WRKY 5	SA signal transduction	Fw: GCATGGTGCAAGAAGTAGGA Rv: CAGCAACAAACGCTACACCT	213 bp	y = −3.41x+30.137	0.99	96.2%	+1.94
ARBRI-C74	bHLH	JA-responsive transcription	Fw: TTACAGCGCAGAATCCCTAA Rv: TGTGCGGGGGAATATAGAAT	213 bp	y = −3.43x+31.282	0.98	95.7%	+1.04
ARBRI-C110	ARF2	Aux-responsive transcription	Fw: ATGTTGGCTCCCGTAGAAAG Rv: ACGAAGGAGGAGGTCCAAC	285 bp	y = −3.36x+30.726	0.99	98.3%	+0.7
ARBRI-C143	GRAS1	Signal transduction defence response	Fw: CGGCGCTCTATATCTTGGAT Rv: ACGTCAAAACGATGGAGTCA	200 bp	y = −3.27x+32.51	1	102%	+1.85
(1) AF545569	*Olea europaea*beta-actin (act1)	Citoskeletal integrity	Fw: GCTTGCTTATGTTGCTCTCGAC Rv: TGATTTCCTTGCTCATACGGTC	308 bp	y = −3.39+29.99	0.99	97%	–

RE values (log_2_ fold-change values) were calculated according to the 2^−ΔΔCt^ method [28]. For all transcripts, RE analysis has been repeated at least two times in independent qRT-PCR experiments (see text for details). Clone ID, gene name, biological process, primers sequences, amplicon length, linear equations, correlation coefficients (R^2^) and PCR efficiencies are also indicated (Fw: Forward; Rv: Reverse). ARBRI = ARBequina Roots Induced gene; ARBRI-C = ARBequina Roots Induced-gene identified as part of a contig. (1): olive act-1 gene used as reference to normalize relative expression (Gene Bank Accession Number).

## Discussion

A functional genomics study was conducted to shed light on the genetic responses taking place during the interaction between a beneficial root endophyte, *P. fluorescens* PICF7, and a woody host such as olive. The bioassay was carried out using non-gnotobiotic experimental conditions aimed to reproduce a near-natural environment. The SSH cDNA library generated in this work thus originated from roots of nursery-produced, potted olive plants growing in a soil-type substrate and carrying their naturally root-associated microbiota. In addition, we have designed a root tissue sampling strategy spanning 21 days after bacterization. This time period is long enough to allow the effective establishment of strain PICF7 on/in olive roots [8], [9], and should therefore enable the identification of olive genes induced during the entire root colonization process by this BCA. Among the approaches that can be implemented to identify genes differentially expressed in response to any given situation (physiological, environmental, etc.), SSH was chosen since it can be performed in the absence of gene sequence information and enables the enrichment of rare transcripts [23]. Moreover, produced cDNA fragments can be directly used for DNA sequencing.

Computational analysis of 445 unique ESTs induced by PICF7 treatment shows a strong up-regulation of transcripts involved, among other processes, in plant defence and response to different kind of stresses. They accounted for up to 44% of the biological processes activated in olive root tissues upon colonization by this beneficial endophyte. This strongly suggests that *P. fluorescens* PICF7 is able to mount an array of defensive responses in olive root tissues. Thus, effective biocontrol activity displayed by PICF7 against VWO [7], [10] could be explained by its ability to activate specific elements involved in early stages of plant defence response processes. Moreover, the ability of PICF7 to internally colonize and persist within olive root tissues indicates that this bacterium is not eventually recognized as a menace by the plant and/or that is able to overcome the host defence response(s) observed in this study.

Results indicated that genes involved in plant hormones and phenylpropanoids biosynthesis (chorismate synthase, PAL, acetone cyanohydrin lyase or lipoxygenase), PR proteins (*β*-1,3-glucanase, endochitinases and lignin-forming peroxidases), and several transcription factors involved in systemic defensive responses (WRKY 5, bHLH, ARF2, GRAS1) are induced in olive roots upon PICF7 colonization. Plant hormones and phenylpropanoids, along with the biosynthesis of alkaloids derived from terpenoids and polyketides, play an important role in (a)biotic stress responses in plants [31]. The induction in bacterized olive roots of the PAL gene transcript, known to be involved in plant defence to pathogens [19], suggests that the PAL response pathway may be activated upon PICF7 colonization thus playing a role in biocontrol activity as similarly reported elsewhere [30], [32], [33].

qRT-PCR validation assays also indicated the induction of lipoxygenase and acetone cyanohydrin lyase genes, known to be involved in the activation of SA- and jasmonate-induced defence pathways [34]. Similarly, stimulation of the lipooxygenase pathway in bean and tomato has been associated with the interaction of *Pseudomonas* spp. strains [35], [36]. On the other hand, the strong up-regulation of the acetone cyanohydrin lyase gene (+2.85 log fold change) after PICF7 bacterization, suggests the induction of an important element of the SA-dependent defence response [37].

However, to properly understand the crosstalk(s) which can be established between different plant defence signalling pathways, the regulatory components (transcription factors) and their capabilities to modulate them [38] should be considered. Indeed, a complex interplay between activating and repressing from multiple classes of transcription factors (ERF, WRKY, Myb, TGA-bZIP and Whirly families) appears to regulate expression of the plant defence transcriptome [39], [40]. In this present study, several transcription factors previously shown to participate in plant defence such as bHLH, GRAS1, ARF2 and SA-responsive WRKY were shown to be induced in olive roots after PICF7 inoculation. Up-to-date, none of these regulatory elements has been identified in olive tissues. In fact, only a limited number of bHLH transcription factors have so far been found involved in plant defence response [41], and very little is known about their role in the modulation of this type of responses. On the other hand, recent studies have provided evidences about the possible function of GRAS proteins as transcriptional activators in the regulation of plant defence responses [42]. The induction of GRAS1 transcription factor expression level here reported could suggest a modulation of olive defence network signalling after PICF7 olive roots colonization. The ARF family of transcription factors regulates a broad range of plant responses to auxin [43], [44]. Up-regulation of ARF2, although at a low level according to validation assays, may suggest the contribution of SAR response in olive upon PICF7 colonization. Moreover, previous physiological and transcriptome studies highlighted the close relationship between auxin and brassinosteroids (BRs), including evidences that promoter regions of BR-responsive genes are enriched in ARF-binding sites [45]. It is tempting to speculate that PICF7 colonization of olive roots might indirectly influence regulation of BR-pathways in olive. Finally, the WRKY 5 transcription factor was identified in this study as induced by PICF7 colonization. Taking into account the complexity of the WRKY family network and the extensive cross-talk among the different signal-transduction pathways involved in plant defence [40], a simultaneous activation of SAR and ISR defence responses by PICF7 in which these regulatory factors play relevant roles cannot be excluded.

This study has also enabled the identification of a large number of new candidate genes such as carbohydrates and ZIP family iron transporters, intrinsic membrane proteins or purine permeases related to cellular communication, potentially participating in this plant-beneficial bacteria interaction. Furthermore, 19% of the olive transcripts induced in roots upon *P. fluorescens* PICF7 colonization corresponded to unidentified genes, stressing the lack of information on functional genomics for olive root tissues and thus the novelty of this work.

**Figure 3:**
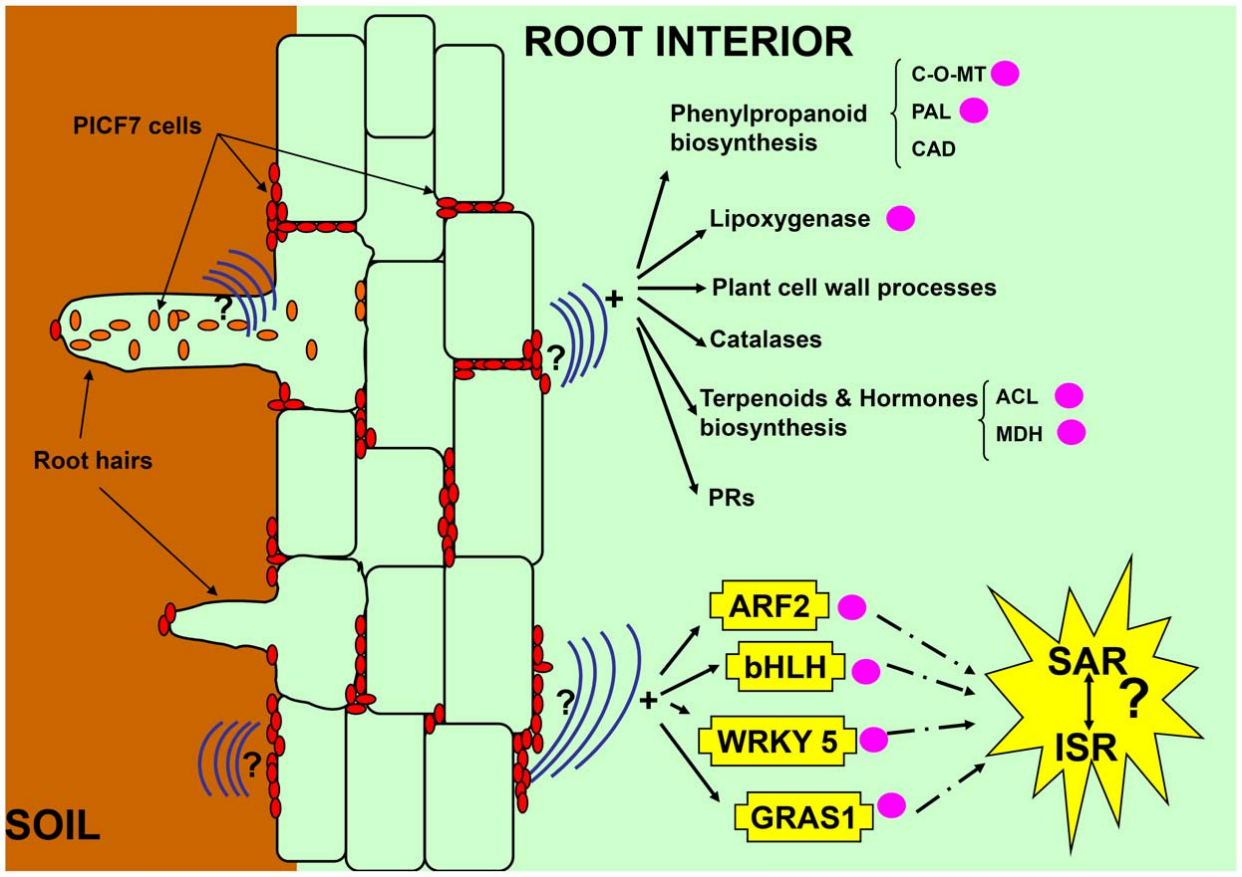
A proposed schematic representation of different defence responses induced in olive root tissues by the beneficial endophyte *Pseudomonas fluorescens* PICF7. An ideal cross section from rhizosphere soil (left) to root cortex (right) is shown. PICF7 cells can effectively colonize the root surface, the intercellular spaces of subepidermal and cortical zones (red ellipses), and the interior of root hairs (orange ellipses) [8], [9]. As yet unidentified signals/metabolites produced by bacteria (represented by curved blue lines) could activate the defence responses here reported (see main text for details). The interaction between *P. fluorescens* PICF7 and olive also induces the expression of different transcription factors involved in systemic acquired resistance (SAR) and induced systemic resistance (ISR), such as bHLH, ARF2 or GRAS1, and WRKYs (i.e., WRKY 5) known to be implicated in the crosstalk between both plant systemic defence response pathways. Genes whose expression has been validated by qRT-PCR analysis are indicated by pink solid circles. C-O-MT: Caffeoyl-O-methyltransferase; PAL: Phenylalanine ammonia-lyase; CAD: Cinnamyl-alcohol dehydrogenase; ACL: Acetone cyanohydrin lyase; MDH: Malate dehydrogenase.

Based in our results, we speculate that PICF7 is able to set off an enhanced protection level in olive roots by activating/modulating an array of defence pathways (Fig. 3). On the other hand, it remains to be elucidated whether genetic responses here observed are the consequence of endophytic colonization by PICF7, or whether they can also be activated before entering into root tissues; that is, during rhizoplane colonization. Endophytic bacteria are able to lessen or prevent the deleterious effects of certain pathogenic organisms. The beneficial effects of bacterial endophytes on their host plant appear to occur through similar mechanisms as described for rhizosphere-associated bacteria and could be associated to induction of resistance [46]. To the best of our knowledge this is the first functional genomics study of an interaction involving a beneficial *P. fluorescens* strain displaying an endophytic lifestyle and a woody host such olive, and may open the way to explore novel strategies to control VWO.

## Supporting Information

Table S1 **List of relevant EST sequences induced in olive roots (cv. Arbequina) by the biocontrol, endophytic strain** ***Pseudomonas fluorescens*** **PICF7.** The EST Sequence Name refers to the codes assigned within the cDNA library. ARBRI means ARBequina Roots Induced gene and ARBRI-C indicates an ARBequina Roots Induced gene identified as part of a Contig. T7 refers to the forward T7 universal primers used for sequencing. Homologous genes were identified in the GenBank protein database (non-redundant) by running the Blastx algorithm set to 1.0 E-3 (in Blast2GO v. 2.4.5). ESTs homologous to protein with unknown function are not included in this table. EST sequence name, putative protein function, organism, accession number, and related E-value are shown.
(DOCX)

Table S2 **List of relevant contigs and their corresponding contiguous/overlapping ESTs.** The EST Sequence Name refers to the codes (User_IDs) found within the cDNA library. ARBRI means ARBequina Roots Induced gene and ARBRI-C indicates an ARBequina Roots Induced gene identified as part of a Contig. T7 refers to the forward T7 universal primers used for sequencing. For more details, see main text and Table S1.
(DOCX)
