# Supplementary material for: Genetic Responses Induced in Olive Roots upon Colonization by the Biocontrol Endophytic Bacterium Pseudomonas fluorescens PICF7
Source: PLoS One. 2012 Nov 7;7(11):e48646. doi: 10.1371/journal.pone.0048646 (PMC3492495; doi:10.1371/journal.pone.0048646)
Supplement: Table S1 — List of relevant EST sequences induced in olive roots (cv. Arbequina) by the biocontrol, endophytic strain Pseudomonas fluorescens PICF7. The EST Sequence Name refers to the codes assigned within the cDNA library. ARBRI means ARBequina Roots Induced gene and ARBRI-C indicates an ARBequina Roots Induced gene identified as part of a Contig. T7 refers to the forward T7 universal primers used for sequencing. Homologous genes were identified in the GenBank protein database (non-redundant) by running the Blastx algorithm set to 1.0 E-3 (in Blast2GO v. 2.4.5). ESTs homologous to protein with unknown function are not included in this table. EST sequence name, putative protein function, organism, accession number, and related E-value are shown. (DOCX) [file pone.0048646.s001.docx]

**Table S1**. **List of relevant EST sequences induced in olive roots (cv. Arbequina) by the biocontrol, endophytic strain *Pseudomonas fluorescens* PICF7.** The EST Sequence Name refers to the codes assigned within the cDNA library. ARBRI means ARBequina Roots Induced gene and ARBRI-C indicates an ARBequina Roots Induced gene identified as part of a Contig. T7 refers to the forward T7 universal primers used for sequencing. Homologous genes were identified in the GenBank protein database (non-redundant) by running the Blastx algorithm set to 1.0 E-3 (in Blast2GO v. 2.4.5). ESTs homologous to protein with unknown function are not included in this table. EST sequence name, putative protein function, organism, accession number, and related E-value are shown.

| **EST Sequence Name** | **Putative protein function** | **Organism** | **Accession Number** | **E-Value** |
| --- | --- | --- | --- | --- |
| ARBRI-C1 | cystein proteinase | *Ipomoea nil* | BAF46302 | 1,41E-65 |
| ARBRI-C3 | eukaryotic initiation factor 5a | *Vitis vinifera* | XP_002284583.1 | 2,03E-28 |
| ARBRI-C5 | skp1-like protein 3 | *Petunia x hybrida* | ACA61785 | 1,75E-47 |
| ARBRI-C6 | short chain alcohol | *Forsythia x intermedia* | AAK38665 | 4,21E-42 |
| ARBRI-C7 | pyruvate decarboxylase | *Citrus sinensis* | AAZ05069 | 5,62E-33 |
| ARBRI-C8 | lipid transfer protein | *Populus trichocarpa* | XP_002330868 | 7,20E-17 |
| ARBRI-C9 | patatin-like protein 1 | *Vitis vinifera* | CBI19560 | 9,23E-66 |
| ARBRI-C11 | papain-like cysteine proteinase | *Ricinus communis* | XP_002512963 | 1,90E-57 |
| ARBRI-C12 | cytochrome p450 | *Solanum chacoense* | P93531 | 2,74E-40 |
| ARBRI-C14 | aldehyde dehydrogenase | *Ricinus communis* | XP_002525343.1 | 8,80E-52 |
| ARBRI-C16 | Dehydrin | *Solanum chilense* | ADQ73953 | 1,73E-16 |
| ARBRI-C17 | ubiquitin-conjugating enzyme e2 | *Brassica napus* | ACC38297 | 9,27E-49 |
| ARBRI-C18 | glutathione transferase | *Vitis vinifera* | CBI27161 | 3,44E-88 |
| ARBRI-C19 | methyl binding domain protein | *Ricinus communis* | XP_002509754 | 1,01E-7 |
| ARBRI-C20 | mta sah | *Populus trichocarpa* | XP_002331152 | 7,85E-77 |
| ARBRI-C21 | aldehyde dehydrogenase family 7 | *Vitis vinifera* | XP_002278093 | 1,23E-16 |
| ARBRI-C22 | 14 kda proline-rich protein | *Vitis vinifera* | XP_002270769 | 7,93E-17 |
| ARBRI-C23 | mip pip subfamily | *Olea europaea* | ABB13429 | 1,52E-14 |
| ARBRI-C24 | hexaubiquitin protein | *Nymphaea hybrid cultivar* | BAJ61942 | 1,51E-30 |
| ARBRI-C25 | thaumatin-like protein | *Sambucus nigra* | AAK59275 | 1,29E-13 |
| ARBRI-C27A | pr-10 protein | *Nicotiana tabacum* | BAJ25784.1 | 4,02E-17 |
| ARBRI-C27C | zip family metal transporter | *Chengiopanax sciadophylloides* | BAE48709 | 2,32E-12 |
| ARBRI-C29 | glutamine synthetase | *Avicennia marina* | AAK08103 | 8,90E-84 |
| ARBRI-C30 | drought-induced protein sdi-6-like | *Olea europaea* | ABS72020 | 4,42E-46 |
| ARBRI-C31 | pathogen induced cyclophilin | *Arachis diogoi* | ABQ53546 | 1,81E-45 |
| ARBRI-C32 | zinc finger protein glo3-like | *Ricinus communis* | XP_002527677 | 3,81E-34 |
| ARBRI-C33 | at3g27090 moj10_18 | *Ricinus communis* | XP_002509516 | 3,86E-71 |
| ARBRI-C34 | vacuolar atp synthase subunit g | *Populus trichocarpa* | XP_002316363 | 2,60E-26 |
| ARBRI-C35 | chorismate synthese | *Solanum Lycopersicum* | Q42884 | 1,20E-51 |
| ARBRI-C36 | serine threonine protein | *Populus trichocarpa* | XP_002312380 | 1,16E-38 |
| ARBRI-C40 | acting on ester | *Vitis vinifera* | XP_002280162 | 1,41E-36 |
| ARBRI-C43 | oligopeptide transporter opt family | *Populus trichocarpa* | XP_002298639 | 1,11E-41 |
| ARBRI-C46 | PLAT_plant_stress | *Vitis vinifera* | CBI31241 | 3,82E-50 |
| ARBRI-C47 | ubiquitin-conjugating enzyme e2-17 kda | *Zea mays* | NP_001146868 | 4,82E-48 |
| ARBRI-C48 | auxin response factor 2 | *Solanum Lycopersicum* | ABC69711 | 4,23E-7 |
| ARBRI-C49 | dimethylmenaquinone methyltransferase | *Gossypium hirsutum* | ACJ11725 | 1,03E-40 |
| ARBRI-C50 | Catalase | *Mesembryanthemum crystallinum* | AAC19397 | 3,38E-67 |
| ARBRI-C51 | malate dehydrogenase | *Glycine max* | ACU17786.1 | 2,31E-68 |
| ARBRI-C52 | superoxide dismutase | *Haberlea rhodopensis* | ADX36104 | 1,02E-18 |
| ARBRI-C53 | cytochrome p450 | *Glycine max* | ABC68413 | 2,23E-46 |
| ARBRI-C54 | pyruvate dehydrogenase | *Citrus x paradis* | AAY86036 | 1,01E-42 |
| ARBRI-C55 | p53 binding protein | *Arabidopsis lyrata* | XP_002888590 | 2,15E-14 |
| ARBRI-C56 | short chain alcohol | *Ricinus communis* | XP_002523859 | 2,28E-31 |

| ARBRI-C58 | aquaporin pip2 | | *Arabidopsis thaliana* | BAA22098 | | 9,99E-44 | |
| --- | --- | --- | --- | --- | --- | --- | --- |
| ARBRI-C60 | short chain alcohol dehydrogenase | | *Solanum tuberosum* | ABA46758 | | 2,38E-28 | |
| ARBRI-C64 | sorbitol transporter | | *Malus x domestica* | BAD42345 | | 5,65E-71 | |
| ARBRI-C65 | u2 snrnp auxiliary small | | *Thellungiella halophila* | BAJ34257 | | 4,32E-6 | |
| ARBRI-C66 | caax prenyl protease | | *Populus trichocarpa* | XP_002302601 | | 7,28E-49 | |
| ARBRI-C67 | cardenolide 16-o-glucohydrolase | | *Olea europaea* | AAL93619 | | 3,06E-23 | |
| ARBRI-C68 | beta-galactosidase | | *Actinidia deliciosa* | ADV41669 | | 1,55E-59 | |
| ARBRI-C70 | Cyclophilin | | *Nicotiana tabacum* | ABS30424 | | 1,33E-50 | |
| ARBRI-C71 | r2r3-myb transcription factor | | *Nicotiana langsdorffii x sandarae* | ABV02032 | | 2,49E-25 | |
| ARBRI-C72 | h+-transporting two-sector | | *Vitis vinifera* | CBI22163 | | 1,17E-51 | |
| ARBRI-C73 | phenylalanine ammonia-lyase | | *Catharanthus roseus* | BAA95629 | | 1,73E-58 | |
| ARBRI-C74 | bhlh transcription factor | | *Solanum Lycopersicum* | AAD46413 | | 9,63E-11 | |
| ARBRI-C75 | ethylene-responsive transcription | | *Vitis vinifera* | XP_002267008 | | 1,90E-41 | |
| ARBRI-C76 | allergen pru protein | | *Salvia miltiorrhiza* | ABR10301 | | 2,12E-32 | |
| ARBRI-C77 | Protein | | *Vitis vinifera* | XP_002272536 | | 3,17E-36 | |
| ARBRI-C78 | short chain alcohol | | *Populus trichocarpa* | XP_002327628 | | 3,57E-66 | |
| ARBRI-C80 | translationally controlled tumor protein | | *Salvia miltiorrhiza* | ABR92336 | | 8,81E-63 | |
| ARBRI-C83 | wrky transcription factor 5 | | *Solanum tuberosum* | ABU49724 | | 6,05E-58 | |
| ARBRI-C84 | xylose isomerise | | *Ricinus communis* | XP_002532409 | | 3,24E-65 | |
| ARBRI-C85 | serine threonine-protein kinase | | *Vitis vinifera* | CBI40719 | | 8,27E-32 | |
| ARBRI-C86 | wrky transcription factor 50-51 | | *Capsicum annuum* | ABP24358 | | 1,42E-26 | |
| ARBRI-C87 | reticuline oxidase precursor | | *Ricinus communis* | XP_002523149 | | 1,89E-49 | |
| ARBRI-C88 | Wound-induced protein 1 | | *Solanum tuberosum* | P20144 | | 2,36E-24 | |
| ARBRI-C89 | glycoside hydrolase family 1 protein | | *Olea europaea* | AAL93619 | | 4,83E-45 | |
| ARBRI-C90 | phosphoglycerate kinase | | *Nicotiana benthamiana* | ADR71054 | | 2,60E-111 | |
| ARBRI-C91 | nuclear transport factor 2 | | *Populus trichocarpa* | XP_002297847 | | 9,13E-57 | |
| ARBRI-C92 | beta-amylase | | *Nicotiana langsdorffii x sanderae* | AAY89374 | | 1,06E-104 | |
| ARBRI-C93 | cold-induced glucosyl transferase | | *Olea europaea* | ACW82415 | | 1,13E-25 | |
| ARBRI-C95 | esterase formylglutathione hydrolase | | *Populus trichocarpa* | XP_002328576 | | 1,12E-33 | |
| ARBRI-C97 | structural constituent of cell wall | | *Ricinus communis* | XP_002517918 | | 5,49E-18 | |
| ARBRI-C99 | receptor protein kinase clavata1 | | *Ricinus communis* | EEF52194 | | 2,90E-9 | |
| ARBRI-C100 | translation initiation factor | | *Carica papaya* | ABS01354 | | 2,10E-29 | |
| ARBRI-C101 | diphthamide biosynthesis protein 3 | | *Ricinus communis* | XP_002519128 | | 3,34E-22 | |
| ARBRI-C102 | superoxide dismutase | | *Haberlea rhodopensis* | ADX36104 | | 6,41E-37 | |
| ARBRI-C103 | cytochrome p450 | | *Vitis vinifera* | CBI30225 | | 8,35E-67 | |
| ARBRI-C104 | hypothetical protein 894764 | | *Arabidopsis lyrata subsp. lyrata* | XP_002888732 | | 1,10E-14 | |
| ARBRI-C105 | short chain alcohol | | *Vitis vinifera* | CBI28275 | | 1,44E-33 | |
| ARBRI-C106 | xri1 (x-ray induced transcript 1) | | *Vitis vinifera* | XP_002272402 | | 2,84E-76 | |
| ARBRI-C107 | sap domain-containing protein | | *Vitis vinifera* | XP_002276745 | | 1,04E-15 | |
| ARBRI-C108 | step ii splicing factor | | *Ricinus communis* | XP_002524613 | | 1,60E-8 | |
| ARBRI-C110 | auxin response factor 2 | | *Solanum Lycopersicum* | ABC69711 | | 4,21E-7 | |
| ARBRI-C111 | acireductone dioxygenase | | *Arabidopsis lyrata* | XP_002868260 | | 1,05E-88 | |
| ARBRI-C112 | 24 kda seed maturation protein | | *Populus trichocarpa* | XP_002299504 | | 3,06E-12 | |
| ARBRI-C113 | subtilisin-like protease | | *Solanum Lycopersicum* | CAA07250 | | 1,38E-12 | |
| ARBRI-C115 | allergen protein | | *Prunus dulcis x persica* | ACE80948 | | 2,77E-10 | |
| ARBRI-C116 | Protein | | *Vitis vinifera* | XP_002283881 | | 2,94E-34 | |
| ARBRI-C117 | glyceraldehyde dehydrogenase | | *Nicotiana tabacum* | CAB39974 | | 1,15E-78 | |
| ARBRI-C118 | protein phosphatise | | *Vitis vinifera* | CBI22956 | | 6,86E-64 | |
| ARBRI-C119 | trehalose 6-phosphate synthase | | *Vitis vinifera* | CBI32125 | | 2,32E-34 | |
| ARBRI-C121 | actin depolymerizing factor 5 | *Populus trichocarpa* | | XP_002305510.1 | 1,15E-45 | |  |
| ARBRI-C122 | pathogenesis-related thaumatin-like | *Theobroma cacao* | | AAV34889 | 5,64E-34 | |  |
| ARBRI-C123 | glycoside hydrolase family 17 protein | *Vitis vinifera* | | XP_002270153 | 6,49E-58 | |  |
| ARBRI-C124 | 2 family protein | *Populus trichocarpa* | | XP_002305805 | 2,21E-21 | |  |
| ARBRI-C125 | conserved hypothetical protein | *Ricinus communis* | | XP_002513455 | 3,38E-11 | |  |
| ARBRI-C126 | apoptosis inhibitory 5 family protein | *Vitis vinifera* | | XP_002282903 | 1,16E-72 | |  |
| ARBRI-C127 | gtp-binding protein | *Ricinus communis* | | XP_002526048 | 7,40E-57 | |  |
| ARBRI-C128 | NDR1/HIN1-Like protein | *Arabidopsis lyrata* | | XP_002862711 | 4,95E-21 | |  |
| ARBRI-C129 | aleurone layer morphogenesis protein | *Vitis vinifera* | | CBI36653 | 1,22E-11 | |  |
| ARBRI-C130 | rhicadhesin receptor | *Ricinus communis* | | XP_002531992 | 3,45E-72 | |  |
| ARBRI-C133 | Enolase | *Glycine max* | | AAS18240 | 1,99E-31 | |  |
| ARBRI-C134 | plasma membrane intrinsic protein | *Olea europaea* | | ABB13429 | 8,15E-77 | |  |
| ARBRI-C135 | translation factor SUI1 protein | *Coffea Arabica* | | CAD58629 | 5,50E-9 | |  |
| ARBRI-C136 | Protein | *Ricinus communis* | | XP_002530876 | 1,85E-42 | |  |
| ARBRI-C137 | atp binding | *Ricinus communis* | | XP_002526287 | 5,19E-12 | |  |
| ARBRI-C138 | c-x8-c-x5-c-x3-h type zn-finger | *Catharanthus roseus* | | P35007 | 1,75E-63 | |  |
| ARBRI-C140 | acetone-cyanohydrin lyase | *Nicotiana tabacum* | | AAR87711 | 1,12E-20 | |  |
| ARBRI-C141 | aldo keto reductase family protein | *Manihot esculenta* | | AAX84672 | 5,78E-14 | |  |
| ARBRI-C143 | GRAS1 transcription factor | *Solanum Lycopersicum* | | ABD72958 | 7,63E-91 | |  |
| ARBRI-C144 | elicitor-responsive protein | *Ricinus communis* | | XP_002533151 | 1,12E-14 | |  |
| ARBRI-C145 | pi starvation-induced protein | *Nicotiana tabacum* | | BAA06151 | 1,45E-25 | |  |
| ARBRI-C146 | short chain alcohol | *Ricinus communis* | | XP_002523859 | 4,20E-31 | |  |
| ARBRI-C147 | alpha-galactosidase | *Salvia miltiorrhiza* | | AAU86897 | 2,23E-50 | |  |
| ARBRI-C151 | lysine ketoglutarate reductase | *Sorghum bicolour* | | XP_002459174 | 2,17E-45 | |  |
| ARBRI-C152 | kunitz trypsin inhibitor 4 | *Glycine max* | | ACU15788 | 3,46E-6 | |  |
| ARBRI-C153 | metal ion binding | *Ricinus communis* | | XP_002509857 | 1,50E-14 | |  |
| ARBRI-C155 | nuclear protein | *Ricinus communis* | | XP_002530606 | 2,83E-29 | |  |
| ARBRI-C156 | RNA and export factor binding protein | *Ricinus communis* | | XP_002531375 | 1,67E-10 | |  |
| ARBRI-C157 | zinc finger domain-containing protein | *Petunia x hybrida* | | AAD02556 | 8,44E-34 | |  |
| ARBRI-C159 | rna binding | *Vitis vinifera* | | XP_002273353 | 1,09E-12 | |  |
| ARBRI-C161 | purine permease | *Vitis vinifera* | | XP_002285717 | 1,42E-29 | |  |
| ARBRI-C164 | kunitz trypsin proteinase inhibitor | *Capsicum annuum* | | P83241 | 9,26E-9 | |  |
| ARBRI-C166 | nicotiana lesion-inducing like | *Nicotiana tabacum* | | AAC49975 | 1,03E-32 | |  |
| ARBRI-C167 | tumor-related protein | *Vitis vinifera* | | CBI35471 | 2,32E-31 | |  |
| ARBRI-C168 | na+ h+ antiporter | *Salicornia europaea* | | AAN08157 | 2,16E-10 | |  |
| ARBRI-C169 | chaperone protein dnaj chloroplast | *Phaseolus vulgaris* | | AAB36543 | 6,27E-21 | |  |
| ARBRI-C171 | beta-galactosidase | *Actinidia deliciosa* | | ADV41669 | 8,04E-14 | |  |
| ARBRI-C173 | flavonol synthase | *Populus trichocarpa* | | XP_002336690 | 1,33E-54 | |  |
| ARBRI-C174 | atp-binding cassette transporter | *Sorghum bicolour* | | XP_002444554 | 4,37E-10 | |  |
| ARBRI-C177 | elav-like protein 4 | *Ricinus communis* | | XP_002530915 | 1,89E-23 | |  |
| ARBRI-C179 | atp citrate lyase | *Populus trichocarpa* | | XP_002312331 | 2,01E-19 | |  |
| ARBRI-C181 | stem-specific protein | *Ricinus communis* | | XP_002519037 | 2,40E-12 | |  |
| ARBRI-C183 | alpha-glucan-protein synthase | *Ricinus communis* | | XP_002512611 | 1,40E-36 | |  |
| ARBRI-C184 | serine hydroxymethyltransferase | *Vitis vinifera* | | CBI17302 | 2,68E-72 | |  |
| ARBRI-C185 | selenoprotein precursor | *Vitis vinifera* | | XP_002273955 | 7,78E-35 | |  |
| ARBRI-C186 | phosphatidyl serine synthase | *Vitis vinifera* | | XP_002283058 | 1,77E-14 | |  |
| ARBRI-C188 | cysteine protease | *Citrus sinensis* | | ABS12459 | 2,54E-38 | |  |
| ARBRI-C189 | transitional endoplasmic reticulum | *Dimocarpus longan* | | ACC66148 | 5,05E-15 | |  |
| ARBRI-C190 | glycosyltransferase-like protein | *Ricinus communis* | | XP_002523710 | 6,48E-18 | |  |
| ARBRI-C191 | Calmodulin | *Zea mays* | | NP_001131288 | 9,58E-54 | |  |
| ARBRI-1_T7_B01 | caffeoyl- o-methyltransferase | *Vitis vinifera* | | AMB79759.1 | 3,50E-56 | |  |
| ARBRI-1_T7_B02 | seed maturation protein | *Ricinus communis* | | XP_002521590 | 1,77E-15 | |  |
| ARBRI-1_T7_C02 | vacuolar atpases | *Citrus limon* | | Q9SWE7 | 4,41E-30 | |  |
| ARBRI-1_T7_D12_ | nac domain ipr003441 | *Ricinus communis* | | XP_002512632 | 4,46E-22 | |  |
| ARBRI-1_T7_E02 | fra e allergen | *Fraxinus excelsior* | | ABU95409 | 1,40E-60 | |  |
| ARBRI-1_T7_E03 | brassinosteroid-regulated protein | *Medicago truncatula* | | ACJ85040 | 8,60E-50 | |  |
| ARBRI-1_T7_E05 | dicarboxylate tricarboxylate carrier | *Citrus junos* | | AAR06239 | 9,56E-25 | |  |
| ARBRI-1_T7_E07 | pathogenesis-related protein 10 | *Vitis vinifera* | | CAN83787 | 9,82E-14 | |  |
| ARBRI-1_T7_E10 | brassinosteroid-regulated protein | *Medicago truncatula* | | ACJ85040 | 8,60E-50 | |  |
| ARBRI-1_T7_E11 | s-adenosylmethionine decarboxylase | *Ipomoea nil]* | | Q96471 | 5,78E-14 | |  |
| ARBRI-1_T7_F06 | cinnamyl alcohol dehydrogenase | *Striga asiatica* | | ABG35772 | 7,80E-65 | |  |
| ARBRI-1_T7_H05 | exocyst complex | *Ricinus communis* | | XP_002511193 | 7,61E-51 | |  |
| ARBRI-1_T7_H06 | 14-3-3 protein | *Manihot esculenta* | | AAY67798 | 7,78E-43 | |  |
| ARBRI-1_T7_H12 | aspartate aminotransferase | *Solanum tuberosum* | | ABB55364 | 1,72E-50 | |  |
| ARBRI-2_T7_B09 | quinone reductase family protei | *Arabidopsis thaliana* | | NP_194457 | 4,52E-35 | |  |
| ARBRI-2_T7_C01 | sorbitol transporter | *Populus trichocarpa* | | XP_002313809 | 2,32E-56 | |  |
| ARBRI-2_T7_C02 | Catalase | *Prunus avium* | | ABM47415 | 1,43E-41 | |  |
| ARBRI-2_T7_C04 | malate dehydrogenase | *Glycine max* | | ACU17786 | 2,34E-68 | |  |
| ARBRI-2_T7_C12 | Catalase | *Oryza sativa Japonica* | | NP_001048861 | 1,43E-51 | |  |
| ARBRI-2_T7_D06 | homeobox-leucine zipper protein | *Vitis vinifera* | | XP_002265944 | 7,62E-11 | |  |
| ARBRI-2_T7_E05 | auxin-repressed protein | *Manihot esculenta* | | AAX84678 | 5,27E-16 | |  |
| ARBRI-2_T7_E06 | calcium-binding protein | *Solanum tuberosum* | | Q09011 | 9,21E-12 | |  |
| ARBRI-2_T7_F05 | Lipoxygenase | *Olea europaea* | | ACG56281.1 | 4,81E-70 | |  |
| ARBRI-2_T7_H04 | f-box family protein | *Populus trichocarpa* | | XP_002307387 | 8,30E-10 | |  |
| ARBRI-2_T7_H05 | beta-glucanase | *Olea europaea* | | CAH17549 | 1,08E-36 | |  |
| ARBRI-2_T7_H08 | protease inhibitor seed storage | *Populus trichocarpa* | | XP_002315191 | 8,53E-26 | |  |
| ARBRI-3_T7_B01 | acyl- synthetase | *Vitis vinifera* | | XP_002270385 | 7,12E-41 | |  |
| ARBRI-3_T7_B06 | trypsin proteinase inhibitor precursor | *Solanum lycopersicum* | | Q43502 | 1,81E-12 | |  |
| ARBRI-3_T7_B12 | aluminum-induced protein | *Solanum tuberosum* | | ABA46788 | 3,41E-19 | |  |
| ARBRI-3_T7_C06 | Dehydrin | *Helianthus petiolaris* | | CAC80712 | 2,71E-8 | |  |
| ARBRI-3_T7_D01 | receptor protein kinase clavata1 | *Populus trichocarpa* | | XP_002325963 | 6,91E-12 | |  |
| ARBRI-3_T7_D04 | peroxidase-like protein | *Vitis vinifera* | | XP_002266365 | 3,37E-58 | |  |
| ARBRI-3_T7_D07 | glutathione s-transferase | *Populus trichocarpa* | | XP_002328824 | 7,16E-49 | |  |
| ARBRI-3_T7_E01 | aluminum-induced protein | *Codonopsis lanceolata]* | | AAW02789.1 | 2,02E-112 | |  |
| ARBRI-3_T7_E08 | phosphate transporter | *Arabidopsis lyrata* | | XP_002879774 | 5,30E-39 | |  |
| ARBRI-3_T7_E12 | phospholipase d | *Gossypium raimondii* | | ACG63795 | 3,01E-59 | |  |
| ARBRI-3_T7_F03 | phosphate synthase | *Vitis vinifera* | | CBI19189 | 7,08E-38 | |  |
| ARBRI-3_T7_G07 | Ubiquitin | *Puccinia graminis* | | EFP86686 | 1,95E-80 | |  |
| ARBRI-3_T7_G08 | translationally controlled tumor prot | *Vitis vinifera* | | XP_002271212 | 1,94E-22 | |  |
| ARBRI-3_T7_H05 | ferredoxin- chloroplast | *Helianthus annuus* | | AAY85661 | 5,05E-32 | |  |
| ARBRI-3_T7_H08 | Cytochrome | *Vitis vinifera* | | XP_002276812 | 2,68E-75 | |  |
| ARBRI-3_T7_H10 | reversibly glycosylated polypeptide-3 | *Vitis vinifera* | | CBI15676 | 1,46E-111 | |  |
| ARBRI-4_T7_A09 | trypsin proteinase inhibitor precursor | *Capsicum annuum* | | P83241 | 2,02E-8 | |  |
| ARBRI-4_T7_B03 | blight-associated protein p12 | *Oryza sativa* | | EAZ09461 | 4,36E-11 | |  |
| ARBRI-4_T7_B06 | beta-galactosidase | *Medicago truncatula* | | ABN08770 | 1,25E-16 | |  |
| ARBRI-4_T7_C05 | aldehyde dehydrogenase | *Fagus sylvatica* | | CBY92007 | 6,11E-24 | |  |
| ARBRI-4_T7_C08 | coated vesicle membrane | *Populus trichocarpa* | | XP_002300383 | 8,84E-12 | |  |
| ARBRI-4_T7_D01 | hexose transporter | *Olea europaea* | | ABJ98314 | 2,05E-80 | |  |
| ARBRI-4_T7_D07 | elongation factor 2 | *Triticum monococcum* | | AAW78583 | 3,5E-26 | |  |
| ARBRI-4_T7_D12 | leucine-rich repeat family protein | *Ricinus communis* | | XP_002521155 | 8,78E-35 | |  |
| ARBRI-4_T7_E07 | ubiquitin-conjugating enzyme e2 i | *Glycine max* | | ACU13836.1 | 3,73E-50 | |  |
| ARBRI-4_T7_F11 | membrane protein | *Vitis vinifera* | | CBI27668 | 1,34E-10 | |  |
| ARBRI-4_T7_G02 | ubiquitin-like protein | *Arabidopsis lyrata* | | XP_002866079 | 2,22E-22 | |  |
| ARBRI-4_T7_G09 | universal stress protein | *Ricinus communis* | | XP_002519217 | 2,13E-53 | |  |
| ARBRI-4_T7_G12 | aldehyde dehydrogenase | *Ricinus communis* | | XP_002518343 | 4,72E-52 | |  |
| ARBRI-4_T7_H03 | Actin | *Actinidia deliciosa* | | ABR45727 | 7,14E-73 | |  |
| ARBRI-4_T7_H08 | beta- glucanase | *Olea europaea* | | CAH17549 | 2,80E-37 | |  |
| ARBRI-4_T7_H10 | HMR1 dna-binding protein | *Antirrhinum majus* | | CAA15421 | 3,82E-16 | |  |
| ARBRI-4_T7_H12 | chemocyanin precursor | *Populus trichocarpa* | | XP_002298184 | 1,91E-26 | |  |
| ARBRI-5_T7_A04 | class IV chitinase | *Vitis vinifera* | | CBI24652 | 7,85E-43 | |  |
| ARBRI-5_T7_A09 | peroxidase-like protein | *Gossypium hirsutum* | | ACJ11762 | 6,24E-57 | |  |
| ARBRI-5_T7_A10 | PGPD14 zinc finger | *Petunia x hybrida* | | AAD02556 | 2,81E-29 | |  |
| ARBRI-5_T7_D07 | atp synthase beta chain | *Plantago major* | | CAJ38391.1 | 6,17E-42 | |  |
| ARBRI-5_T7_D09 | phosphate synthase | *Populus trichocarpa* | | XP_002307364 | 5,24E-42 | |  |
| ARBRI-5_T7_F05 | lignin-forming anionic peroxidase | *Nicotiana sylvestris* | | Q02200 | 1,14E-33 | |  |
| ARBRI-5_T7_G05 | beta xylosidase | *Camellia sinensis* | | ACD93208 | 1,26e-45 | |  |
| ARBRI-5_T7_H07 | beta-catenin-like protein 1 | *Ricinus communis* | | XP_002526189 | 2,74E-24 | |  |
| ARBRI-6_T7_A01 | nadp-malic enzyme | *Solanum lycopersicum* | | AAB58727 | 2,75E-42 | |  |
| ARBRI-6_T7_A10 | phosphoenolpyruvate carboxylase | *Panicum ovuliferum* | | CAM84112 | 4,22E-25 | |  |
| ARBRI-6_T7_B02 | cytochrome p450 | *Vitis vinifera* | | CBI19610 | 1,54E-35 | |  |
| ARBRI-6_T7_B12 | glutathione s-transferase | *Jatropha curcas* | | ADB85090 | 6,94E-20 | |  |
| ARBRI-6_T7_C01 | phloem transcription factor m1 | *Populus trichocarpa* | | XP_002315956 | 2,30E-7 | |  |
| ARBRI-6_T7_C11 | gdp-mannose pyrophosphorylase | *Carica papaya* | | ACN66754 | 1,37E-15 | |  |
| ARBRI-6_T7_D01 | ornithine aminotransferase | *Nicotiana tabacum* | | ADM47437 | 6,43E-21 | |  |
| ARBRI-6_T7_D03 | d-3-phosphoglycerate dehydrogenas | *Ricinus communis* | | XP_002518687 | 2,33E-106 | |  |
| ARBRI-6_T7_D05 | plasma membrane intrinsic protein 1a | *Fraxinus excelsior* | | AAT74898 | 1,00E-18 | |  |
| ARBRI-6_T7_E02 | glutamate decarboxylase | *Ricinus communis* | | XP_002528515 | 2,09E-40 | |  |
| ARBRI-6_T7_E05 | abscisic insensitive 1b | *Solanum lycopersicum* | | BAI39595 | 8,70E-34 | |  |
| ARBRI-6_T7_F12 | protein phosphatise | *Vitis vinifera* | | CBI22956 | 2,63E-63 | |  |
| ARBRI-6_T7_H05 | short chain alcohol | *Forsythia x intermedia* | | AAK38665 | 3,40E-38 | |  |
| ARBRI-6_T7_H06 | diphosphate reductase | *Catharanthus roseus* | | ABI30631 | 1,85E-36 | |  |
| ARBRI-7_T7_A02 | superoxide dismutase | *Fagus sylvatica* | | CAE54085 | 3,45E-72 | |  |
| ARBRI-7_T7_A05 | plant viral-response family | *Vitis vinifera* | | XP_002284765 | 1,08E-67 | |  |
| ARBRI-7_T7_A07 | sorbitol transporter | *Populus trichocarpa* | | XP_002313809 | 2,28E-56 | |  |
| ARBRI-7_T7_A08 | cysteine protease | *Ipomoea nil* | | BAF46302 | 3,03E-14 | |  |
| ARBRI-7_T7_B02 | cytochrome p450 | *Vitis vinifera* | | CBI30225 | 1,15E-60 | |  |
| ARBRI-7_T7_C08 | 14 kda proline-rich protein | *Vitis vinifera* | | XP_002270769 | 1,36E-21 | |  |
| ARBRI-7_T7_C11 | radical-induced cell death1 protein | *Vitis vinifera* | | CBI33838 | 3,77E-17 | |  |
| ARBRI-7_T7_D03 | 33 kda secretory protein | *Ricinus communis]* | | XP_002533056 | 1,09E-36 | |  |
| ARBRI-7_T7_E04 | pathogen-related protein | *Salvia miltiorrhiza* | | ABR10301 | 1,07E-7 | |  |
| ARBRI-7_T7_F05 | bzip domain class transcription factor | *Populus trichocarpa* | | XP_002306888 | 4,13E-12 | |  |
| ARBRI-7_T7_F06 | pathogenesis-related thaumatin | *Vitis vinifera* | | XP_002274137 | 6,07E-8 | |  |
| ARBRI-7_T7_F09 | small gtp-binding protein | *Ricinus communis* | | XP_002531780 | 1,05E-19 | |  |
| ARBRI-7_T7_F12 | Aquaporin | *Iris x hollandica* | | BAF44223 | 1,14E-38 | |  |
| ARBRI-7_T7_G01 | ornithine aminotransferase | *Vitis vinifera* | | CAN73779 | 2,58E-12 | |  |
| ARBRI-7_T7_G04 | phosphoric diester hydrolase | *Ricinus communis* | | XP_002516864 | 3,04E-39 | |  |
| ARBRI-7_T7_H04 | zinc finger | *Medicago truncatula* | | ABN08073 | 1,02E-8 | |  |
| ARBRI-7_T7_H08 | Glutaredoxin | *Tilia platyphyllos* | | AAL04507 | 5,34E-38 | |  |
| ARBRI-8_T7_A03 | r3h domain containing | *Vitis vinifera* | | XP_002283240 | 1,30E-15 | |  |
| ARBRI-8_T7_A05 | auxin signaling f-box 3 | *Populus trichocarpa* | | XP_002328871 | 5,48E-20 | |  |
| ARBRI-8_T7_B03 | GRAS1 | *Solanum lycopersicum* | | ABD72958 | 1,6E-4 | |  |
| ARBRI-8_T7_B04 | cytoplasmic aconitate hydratase | *Citrus clementina* | | CBE71057 | 2,42E-65 | |  |
| ARBRI-8_T7_B05 | clathrin coat assembly protein | *Populus trichocarpa* | | XP_002318532 | 1,03E-10 | |  |
| ARBRI-8_T7_B12 | auxin-induced protein | *Ricinus communis* | | XP_002531394 | 4,83E-29 | |  |
| ARBRI-8_T7_D03 | ubiquitin-protein ligase | *Ricinus communis* | | XP_002510699 | 2,04E-4 | |  |
| ARBRI-8_T7_D06 | cysteine proteinase | *Narcissus pseudonarcissus* | | AAL69389 | 1,05E-9 | |  |
| ARBRI-8_T7_D07 | ef hand family protein | *Populus trichocarpa* | | XP_002319225 | 4,0E-10 | |  |
| ARBRI-8_T7_F07 | Tomf2 | *Nicotiana tomentosiformis* | | AAZ81601 | 1,43E-25 | |  |
| ARBRI-8_T7_F10 | nucleic acid binding | *Vitis vinifera* | | XP_002283240 | 5,61E-33 | |  |
| ARBRI-8_T7_G02 | protein kinase c inhibitor | *Ricinus communis* | | XP_002520586 | 1,48E-44 | |  |
| ARBRI-8_T7_G05 | pyruvate dehydrogenase | *Populus trichocarp* | | XP_002311788 | 3,57E-42 | |  |
| ARBRI-8_T7_G06 | Glycoprotein | *Populus trichocarpa* | | XP_002306876 | 1,66E-13 | |  |
| ARBRI-8_T7_H05 | beta-galactosidase | *Actinidia deliciosa* | | ADV41669 | 1,64E-19 | |  |
| ARBRI-8_T7_H08 | ubiquitin-protein ligase | *Ricinus communis* | | XP_002510699 | 3,04E-23 | |  |
| ARBRI-8_T7_H09 | armadillo beta-catenin | *Oryza sativa Japonica* | | NP_001176902 | 4,06E-8 | |  |
| ARBRI-9_T7_A03 | f-box family expressed | *Vitis vinifera* | | CBI31833 | 1,01E-9 | |  |
| ARBRI-9_T7_A10 | phosphoglycerate mutase | *Mesembryanthemum crystallinum* | | Q42908 | 1,83E-60 | |  |
| ARBRI-9_T7_B02 | dehydration responsive element | *Arabidopsis lyrata* | | XP_002878679 | 4,87E-10 | |  |
| ARBRI-9_T7_D02 | acyl carrier protein | *Arachis hypogaea* | | ACZ06073 | 6,40E-29 | |  |
| ARBRI-9_T7_E04 | apyrase-like protein | *Olea europaea* | | ABS72007 | 5,30E-67 | |  |
| ARBRI-9_T7_E07 | metallopeptidase family m24 | *Vitis vinifera* | | XP_002273246 | 5,64E-46 | |  |
| ARBRI-9_T7_F10 | mta sah | *Vitis vinifera* | | CBI28039 | 5,47E-62 | |  |
| ARBRI-9_T7_F11 | stem-specific protein | *Vitis vinifera* | | CBI41052 | 9,64E-18 | |  |
| ARBRI-9_T7_F12 | exostosin-like glycosyltransferase | *Ricinus communis* | | XP_002526728 | 9,38E-57 | |  |
| ARBRI-9_T7_G01 | adaptin ear-binding coat-associated | *Ricinus communis* | | XP_002525119 | 3,72E-21 | |  |
| ARBRI-9_T7_G08 | receptor-like protein kinase | *Glycine max* | | ACM89557 | 4,08E-10 | |  |
| ARBRI-9_T7_H06 | thioredoxin h | *Salvia miltiorrhiza* | | ACI31202 | 7,71E-11 | |  |
| ARBRI-10_T7_A06 | chitinase-like protein | *Vitis vinifera* | | CBI22907 | 9,10E-43 | |  |
| ARBRI-10_T7_A08 | aspartic protease precursor-like | *Solanum tuberosum* | | ABB87123 | 2,13E-40 | |  |
| ARBRI-10_T7_A11 | diphosphoinositol polyphosphate | *Glycine max* | | ACU19981 | 2,02E-19 | |  |
| ARBRI-10_T7_A12 | spermidine synthase | *Solanum lycopersicum* | | AAP97136 | 5,50E-30 | |  |
| ARBRI-10_T7_B01 | Catalase | *Prunus persica* | | CAB56850 | 5,94E-61 | |  |
| ARBRI-10_T7_B05 | Peroxidise | *Populus trichocarpa* | | XP_002322726 | 7,36E-66 | |  |
| ARBRI-10_T7_B11 | chitinase A | *Ananas comosu* | | BAG38685 | 8,18E-53 | |  |
| ARBRI-10_T7_C03 | Protein HVA22, putative | *Ricinus communis* | | XP_002511560 | 8,90E-68 | |  |
| ARBRI-10_T7_C06 | 14 kda proline-rich protein | *Vitis vinifera* | | XP_002270839 | 4,04E-14 | |  |
| ARBRI-10_T7_C08 | translation factor | *Oryza sativa Japonica* | | EEE67305 | 6,01E-8 | |  |
| ARBRI-10_T7_D01 | ap2 domain class transcription factor | *Nicotiana tabacum* | | AAP40022 | 1,21E-35 | |  |
| ARBRI-10_T7_D02 | LEA_3 | *Arabidopsis thaliana* | | AAC19273.1 | 1,51E-19 | |  |
| ARBRI-10_T7_D06 | tubulin-specific chaperone a | *Ricinus communis* | | XP_002523979 | 1,09E-6 | |  |
| ARBRI-10_T7_E01 | plasma membrane intrinsic protein | *Olea europaea* | | ABB13430 | 1,59E-48 | |  |
| ARBRI-10_T7_E11 | serine threonine-protein kinase | *Vitis vinifera* | | CBI40719 | 8,07E-32 | |  |
| ARBRI-10_T7_E12 | stachyose synthase | *Vitis vinifera* | | XP_002275829 | 2,15E-47 | |  |
| ARBRI-10_T7_F01 | aldo keto reductase family protein | *Arabidopsis thaliana* | | 3H7U_A | 3,47E-35 | |  |
| ARBRI-10_T7_F05 | choline kinase 2p-like protein | *Ricinus communis* | | XP_002524355 | 1,99E-22 | |  |
| ARBRI-10_T7_F06 | ran-binding protein 1 | *Populus trichocarpa* | | XP_002299634 | 3,28E-33 | |  |
| ARBRI-10_T7_F09 | malate dehydrogenase (nadp+) | *Vitis vinifera* | | CBI20568 | 1,69E-110 | |  |
| ARBRI-10_T7_F12 | aldehyde dehydrogenase | *Populus trichocarpa* | | XP_002318034 | 6,86E-64 | |  |
| ARBRI-10_T7_G05 | pyruvate dehydrogenase | *Citrus x paradisi* | | AAY86036 | 1,64E-24 | |  |
| ARBRI-10_T7_H01 | serine threonine kinase | *Vitis vinifera* | | CBI37539 | 4,38E-22 | |  |
| ARBRI-10_T7_H04 | leucine-rich, serine-threonine kinase | *Ricinus communis* | | XP_002530200 | 1,31E-34 | |  |
| ARBRI-10_T7_H12 | stem-specific protein | *Ricinus communis* | | XP_002519037 | 6,74E-15 | |  |
