# Supplementary material for: Genetic Responses Induced in Olive Roots upon Colonization by the Biocontrol Endophytic Bacterium Pseudomonas fluorescens PICF7
Source: PLoS One. 2012 Nov 7;7(11):e48646. doi: 10.1371/journal.pone.0048646 (PMC3492495; doi:10.1371/journal.pone.0048646)
Supplement: Table S2 — List of relevant contigs and their corresponding contiguous/overlapping ESTs. The EST Sequence Name refers to the codes (User_IDs) found within the cDNA library. ARBRI means ARBequina Roots Induced gene and ARBRI-C indicates an ARBequina Roots Induced gene identified as part of a Contig. T7 refers to the forward T7 universal primers used for sequencing. For more details, see main text and Table S1. (DOCX) [file pone.0048646.s002.docx]

**Table S2**. **List of relevant contigs and their corresponding contiguous/overlapping ESTs.** The EST Sequence Name refers to the codes (User_IDs) found within the cDNA library. ARBRI means ARBequina Roots Induced gene and ARBRI-C indicates an ARBequina Roots Induced gene identified as part of a Contig. T7 refers to the forward T7 universal primers used for sequencing. For more details, see main text and Table S1.

| **Contig names used in this study** | **EST sequences names (User_IDs) as found in dbEST/dbGSS/dbSTS databases** |
| --- | --- |
| ARBRI-C1 | ARBRI-1_T7_A12; ARBRI-1_T7_B09; ARBRI-1_T7_A02; ARBR1_T7_B06; ARBRI-4_T7_B03; ARBRI-4_T7_G11; ARBRI-5_T7_A03; ARBRI-5_T7_E09; ARBRI-7_T7_F04; ARBRI-9_T7_B11 |
| ARBRI-C3 | ARBRI-1_T7_A05; ARBRI4_T7_D10; ARBRI-9_T7_G04; ARBRI-10_T7_F08 |
| ARBRI-C5 | ARBRI-1_T7_A07; ARBRI-6_T7_C02 |
| ARBRI-C6 | ARBRI-1_T7_A09; ARBRI-5_T7_A07; ARBRI-2_T7_B06; ARBRI-3_T7_E11; ARBRI-6_T7_D08; ARBRI-8_T7_D01; ARBRI-9_T7_C02; ARBRI-7_T7_F11; ARBRI-9_T7_B04; ARBRI-9_T7_F04; ARBRI-5_T7_F03; ARBRI-5_T7_H06; ARBRI-5_T7_E11; ARBRI-7_T7_C12; ARBRI-10_T7_E09 |
| ARBRI-C7 | ARBRI-1_T7_A10; ARBRI-3_T7_B03; ARBRI-4_T7_A02; ARBRI-10_T7_G03 |
| ARBRI-C8 | ARBRI-1_T7_B03; ARBRI-2_T7_F09; ARBRI-8_T7_H01 |
| ARBRI-C9 | ARBRI-1_T7_B04; ARBRI-3_T7_B09 |
| ARBRI-C11 | ARBRI-1_T7_B08; ARBRI-4_T7_E06; ARBRI-2_T7_G08; ARBRI-3_T7_D11; ARBRI-4_T7_A04; ARBRI-5_T7_G04; ARBRI-4_T7_F01; ARBRI-5_T7_F02; ARBRI-8_T7_E10; ARBRI-8_T7_H11; ARBRI-10_T7_H10; ARBRI-7_T7_B09 |
| ARBRI-C12 | ARBRI1_T7_C01; ARBRI-1_T7_D01; ARBRI-4_T7_B02; ARBRI-9_T7_C01; ARBRI-7_T7_A04; ARBRI-8_T7_B01 |
| ARBRI-C14 | ARBRI-1_T7_C04; ARBRI-1_T7_C06 |
| ARBRI-C16 | ARBRI-1_T7_C07; ARBRI-4_T7_H07; ARBRI-10_T7_D10; ARBRI-7_T7_D05 |
| ARBRI-C17 | ARBRI-1_T7_C09; ARBRI-1_T7_H11 |
| ARBRI-C18 | ARBRI-1_T7_C11; ARBRI-3_T7_G03; ARBRI-7_T7_A09; ARBRI-9_T7_F07 |
| ARBRI-C19 | ARBRI-1_T7_D02; ARBRI-3_T7_D09 |
| ARBRI-C20 | ARBRI-1_T7_D03; ARBRI-6_T7_F07; ARBRI-7_T7_C01 |
| ARBRI-C21 | ARBRI-1_T7_D04; ARBRI-10_T7_A07 |
| ARBRI-C22 | ARBRI-1_T7_D07; ARBRI-9_T7_A08; ARBRI4_T7_B10; ARBRI-6_T7_H10; ARBRI-9_T7_B03; ARBRI-6_T7_F03; ARBRI-7_T7_F03 |
| ARBRI-C23 | ARBRI-1_T7_D09; ARBRI-3_T7_E09; ARBRI-4_T7_A05 |
| ARBRI-C24 | ARBRI-1_T7_D10; ARBRI-7_T7_G07; ARBRI-9_T7_G07 |
| ARBRI-C25 | ARBRI-1_T7_D11; ARBRI-5_T7_B09; ARBRI-2_T7_C10; ARBRI-2_T7_D04; ARBRI-5_T7_F07; ARBRI-8_T7_G04; ARBRI-6_T7_C07; ARBRI-7_T7_B10; ARBRI-10_T7_D11; ARBRI-8_T7_H03; ARBRI-7_T7_A10; ARBRI-9_T7_C04; ARBRI-8_T7_E05; ARBRI-3_T7_C02; ARBRI-1_T7_F03; ARBRI-2_T7_F08; ARBRI-6_T7_B11; ARBRI-1_T7_E09; ARBRI-10_T7_B02 |
| ARBRI-C27A | ARBRI-1_T7_E04; ARBRI-2_T7_D03; ARBRI-8_T7_A07 |
| ARBRI-C27C | ARBRI-1_T7_E06; ARBRI-5_T7_B10; ARBRI-9_T7_H01 |
| ARBRI-C29 | ARBRI-1_T7_E08; ARBRI-7_T7_B04 |
| ARBRI-C30 | ARBRI-1_T7_F02; ARBRI-2_T7_E10; ARBRI-9_T7_F09; ARBRI-7_T7_G11; ARBRI-4_T7_D09; ARBRI-6_T7_E08 |
| ARBRI-C31 | ARBRI-1_T7_F05; ARBRI-7_T7_H06 |
| ARBRI-C32 | ARBRI-1_T7_F07; ARBRI-7_T7_G08 |
| ARBRI-C33 | ARBRI-1_T7_F08; ARBRI-8_T7_A09 |
| ARBRI-C34 | ARBRI-1_T7_F09; ARBRI-2_T7_B01; ARBRI-7_T7_H07; ARBRI-10_T7_G08 |
| ARBRI-C35 | ARBRI-1_T7_F10; ARBRI-6_T7_D12; ARBRI-8_T7_B07; ARBRI-10_T7_E05 |
| ARBRI-C36 | ARBRI-1_T7_F11; ARBRI-5_T7_C08; ARBRI-7_T7_B11; ARBRI-8_T7_C07 |
| ARBRI-C40 | ARBRI-1_T7_G04; ARBRI-2_T7_D08; ARBRI-5_T7_F12 |
| ARBRI-C43 | ARBRI-1_T7_G08; ARBRI-3_T7_G02; ARBRI-7_T7_D07 |
| ARBRI-C46 | ARBRI-1_T7_H01; ARBRI-5_T7_E06; ARBRI-6_T7_A11; ARBRI-9_T7_D10; ARBRI-9_T7_C03 |
| ARBRI-C47 | ARBRI-1_T7_H03; ARBRI-2_T7_E09 |
| ARBRI-C48 | ARBRI-1_T7_H09; ARBRI-3_T7_A01; ARBRI-7_T7_E11 |
| ARBRI-C49 | ARBRI-1_T7_H10; ARBRI-3_T7_E10; ARBRI-7_T7_D10 |
| ARBRI-C50 | ARBRI-2_T7_A01; ARBRI-2_T7_G04; ARBRI-3_T7_F02; ARBRI-3_T7_H02; ARBRI-3_T7_E02 |
| ARBRI-C51 | ARBRI-2_T7_A04; ARBRI-7_T7_H10 |
| ARBRI-C52 | ARBRI-2_T7_A09; ARBRI-4_T7_D04; ARBRI-8_T7_A01; ARBRI-10_T7_B07; ARBRI-6_T7_B01; ARBRI-6_T7_C04; ARBRI-10_T7_B03 |
| ARBRI-C53 | ARBRI-2_T7_A09; ARBRI-4_T7_D04; ARBRI-8_T7_A01; ARBRI-10_T7_B07; ARBRI-6_T7_B01; ARBRI-6_T7_C04; ARBRI-10_T7_B03 |
| ARBRI-C54 | ARBRI-2_T7_A09; ARBRI-4_T7_D04; ARBRI-8_T7_A01; ARBRI-10_T7_B07; ARBRI-6_T7_B01; ARBRI-6_T7_C04; ARBRI-10_T7_B03 |
| ARBRI-C55 | ARBRI-2_T7_A08; ARBRI-2_T7_B08 |
| ARBRI-C56 | ARBRI-2_T7_A09; ARBRI-4_T7_D04; ARBRI-8_T7_A01; ARBRI-10_T7_B07; ARBRI-6_T7_B01; ARBRI-6_T7_C04; ARBRI-10_T7_B03 |
| ARBRI-C58 | ARBRI-2_T7_A12; ARBRI-2_T7_G11; ARBRI-3_T7_A02; ARBRI-3_T7_B05; ARBRI-6_T7_D04 |
| ARBRI-C60 | ARBRI-2_T7_B03; ARBRI-6_T7_E01 |
| ARBRI-C64 | ARBRI-2_T7_C01; ARBRI-4_T7_E02; ARBRI-5_T7_B04; ARBRI-7_T7_A07 |
| ARBRI-C65 | ARBRI-2_T7_C03; ARBRI-5_T7_H08 |
| ARBRI-C66 | ARBRI-2_T7_C07; ARBRI-3_T7_A03; ARBRI-6_T7_D09 |
| ARBRI-C67 | ARBRI-2_T7_C08; ARBRI-8_T7_G07 |
| ARBRI-C68 | ARBRI-2_T7_C09; ARBRI-2_T7_D07; ARBRI-5_T7_H09; ARBRI-5_T7_B05; ARBRI-8_T7_B10; ARBRI-10_T7_A09; ARBRI-8_T7_C10 |
| ARBRI-C70 | ARBRI-2_T7_D05; ARBRI-5_T7_C10; ARBRI-9_T7_D03 |
| ARBRI-C71 | ARBRI-2_T7_D09; ARBRI-3_T7_E07; ARBRI-5_T7_C04; ARBRI-10_T7_C07; ARBRI-9_T7_B05; ARBRI-9_T7_B10 |
| ARBRI-C72 | ARBRI-2_T7_D10; ARBRI-5_T7_G08; ARBRI-9_T7_D06 |
| ARBRI-C73 | ARBRI-2_T7_E01; ARBRI-2_T7_F01; ARBRI-5_T7_D04 |
| ARBRI-C74 | ARBRI-2_T7_E02; ARBRI-4_T7_A08; ARBRI-8_T7_B11 |
| ARBRI-C75 | ARBRI-2_T7_E03; ARBRI-4_T7_G04; ARBRI-5_T7_C06; ARBRI-6_T7_G10 |
| ARBRI-C76 | ARBRI-2_T7_H11; ARBRI-4_T7_F04; ARBRI-4_T7_F03; ARBRI-6_T7_B05; ARBRI-7_T7_C07; ARBRI-2_T7_E07; ARBRI-3_T7_A11; ARBRI-4_T7_B01; ARBRI-7_T7_E07; ARBRI-5_T7_C05; ARBRI-7_T7_D12; ARBRI-3_T7_D06; ARBRI-10_T7_H11; ARBRI-7_T7_H12 |
| ARBRI-C77 | ARBRI-2_T7_E08; ARBRI-4_T7_F017; ARBRI-6_T7_A09; ARBRI-7_T7_D08; ARBRI-8_T7_H12 |
| ARBRI-C78 | ARBRI-2_T7_E12; ARBRI-7_T7_E12; ARBRI-5_T7_B01; ARBRI-7_T7_C04; ARBRI-5_T7_B03; ARBRI-9_T7_A11; ARBRI-9_T7_A09; ARBRI-10_T7_H02 |
| ARBRI-C80 | ARBRI-4_T7_A08; ARBRI-10_T7_D03; ARBRI-9_T7_D08; ARBRI-2_T7_F03; ARBRI-3_T7_G11 |
| ARBRI-C83 | ARBRI-2_T7_F07; ARBRI-4_T7_E08 |
| ARBRI-C84 | ARBRI-2_T7_F10; ARBRI-6_T7_E10 |
| ARBRI-C85 | ARBRI-2_T7_F11; ARBRI-10_T7_E11 |
| ARBRI-C86 | ARBRI-2_T7_G01; ARBRI-8_T7_F04 |
| ARBRI-C87 | ARBRI-2_T7_H09; ARBRI-7_T7_F07; ARBRI-6_T7_C10; ARBRI-6_T7_E03; ARBRI-9_T7_F06; ARBRI-9_T7_H11; ARBRI-8_T7_D08; ARBRI-2_T7_G07; ARBRI-2_T7_G03; ARBRI-9_T7_A02; ARBRI-8_T7_E09; ARBRI-9_T7_A07 |
| ARBRI-C88 | ARBRI-2_T7_G06; ARBRI-5_T7_C07; ARBRI-6_T7_E09 |
| ARBRI-C89 | ARBRI-2_T7_G10; ARBRI-9_T7_D07; ARBRI-8_T7_C09 |
| ARBRI-C90 | ARBRI-2_T7_G12; ARBRI-8_T7_G12 |
| ARBRI-C91 | ARBRI-2_T7_H02; ARBRI-6_T7_B08 |
| ARBRI-C92 | ARBRI-2_T7_H03; ARBRI-10_T7_A03; ARBRI-3_T7_A09; ARBRI-3_T7_C10 |
| ARBRI-C93 | ARBRI-2_T7_H06; ARBRI-3_T7_A12; ARBRI-6_T7_F05 |
| ARBRI-C95 | ARBRI-3_T7_A05; ARBRI-10_T7_B04 |
| ARBRI-C97 | ARBRI-3_T7_A08; ARBRI-7_T7_B12 |
| ARBRI-C99 | ARBRI-3_T7_B02; ARBRI-4_T7_C12 |
| ARBRI-C100 | ARBRI-3_T7_B04; ARBRI-7_T7_G06 |
| ARBRI-C101 | ARBRI-3_T7_B07; ARBRI-4_T7_C09 |
| ARBRI-C102 | ARBRI-3_T7_B11; ARBRI-4_T7_E05 |
| ARBRI-C103 | ARBRI-3_T7_C09; ARBRI-5_T7_A02; ARBRI-8_T7_B09; ARBRI-8_T7_C01; ARBRI-10_T7_C02; ARBRI-3_T7_C01 |
| ARBRI-C104 | ARBRI-3_T7_C07; ARBRI-7_T7_A11 |
| ARBRI-C105 | ARBRI-3_T7_C08; ARBRI-4_T7_D06; ARBRI-6_T7_H12; ARBRI-9_T7_B09 |
| ARBRI-C106 | ARBRI-3_T7_C11; ARBRI-4_T7_D05; ARBRI-4_T7_E12 |
| ARBRI-C107 | ARBRI-3_T7_C12; ARBRI-4_T7_B04; ARBRI-5_T7_A12 |
| ARBRI-C108 | ARBRI-3_T7_D02; ARBRI-4_T7_C07; ARBRI-10_T7_D08 |
| ARBRI-C110 | ARBRI-3_T7_D05; ARBRI-7_T7_C03 |
| ARBRI-C111 | ARBRI-3_T7_D08; ARBRI-10_T7_A01; ARBRI-10_T7_F04 |
| ARBRI-C112 | ARBRI-3_T7_D10; ARBRI-10_T7_H06 |
| ARBRI-C113 | ARBRI-3_T7_E04; ARBRI-9_T7_H05; ARBRI-6_T7_G06 |
| ARBRI-C115 | ARBRI-3_T7_F04; ARBRI-3_T7_F07; ARBRI-3_T7_H06; ARBRI-7_T7_G03; ARBRI-8_T7_D04; ARBRI-9_T7_F02; ARBRI-10_T7_G11; ARBRI-6_T7_H01 |
| ARBRI-C116 | ARBRI-3_T7_F06; ARBRI-3_T7_G10 |
| ARBRI-C117 | ARBRI-3_T7_F08; ARBRI-7_T7_D04; ARBRI-7_T7_E02; ARBRI-7_T7_G09 |
| ARBRI-C118 | ARBRI-3_T7_F10; ARBRI-5_T7_D11; ARBRI-5_T7_G10; ARBRI-6_T7_F12 |
| ARBRI-C119 | ARBRI-3_T7_F11; ARBRI-10_T7_E04 |
| ARBRI-C121 | ARBRI-3_T7_G04; ARBRI-6_T7_H11 |
| ARBRI-C122 | ARBRI-3_T7_G05; ARBRI-5_T7_C09; ARBRI-5_T7_D12; ARBRI-10_T7_B10; ARBRI-10_T7_D05 |
| ARBRI-C123 | ARBRI-3_T7_G06; ARBRI-4_T7_F06; ARBRI-4_T7_F09; ARBRI-7_T7_C02 |
| ARBRI-C124 | ARBRI-3_T7_G09; ARBRI-8_T7_G10 |
| ARBRI-C125 | ARBRI-3_T7_H03; ARBRI-5_T7_E12 |
| ARBRI-C126 | ARBRI-3_T7_H04; ARBRI-4_T7_C10 |
| ARBRI-C127 | ARBRI-3_T7_H07; ARBRI-4_T7_C01 |
| ARBRI-C128 | ARBRI-3_T7_H11; ARBRI-6_T7_D11; ARBRI-8_T7_D02; ARBRI-9_T7_B12; ARBRI-9_T7_H09 |
| ARBRI-C129 | ARBRI-4_T7_A01; ARBRI-8_T7_F01 |
| ARBRI-C130 | ARBRI-4_T7_A03; ARBRI-4_T7_E01; ARBRI-4_T7_G03; ARBRI-8_T7_C11; ARBRI-6_T7_G09; ARBRI-7_T7_E03 |
| ARBRI-C133 | ARBRI-4_T7_A11; ARBRI-4_T7_C03; ARBRI-5_T7_G11; ARBRI-10_T7_B08 |
| ARBRI-C134 | ARBRI-4_T7_A12; ARBRI-8_T7_H06 |
| ARBRI-C135 | ARBRI-4_T7_B05; ARBRI-10_T7_D07 |
| ARBRI-C136 | ARBRI-4_T7_B07; ARBRI-5_T7_H10; ARBRI-9_T7_F01 |
| ARBRI-C137 | ARBRI-4_T7_B09; ARBRI-5_T7_D02 |
| ARBRI-C138 | ARBRI-4_T7_C02; ARBRI-9_T7_D04 |
| ARBRI-C140 | ARBRI-4_T7_C06; ARBRI-9_T7_G02; ARBRI-5_T7_F06; ARBRI-8_T7_F09 |
| ARBRI-C141 | ARBRI-4_T7_C11; ARBRI-9_T7_A04 |
| ARBRI-C143 | ARBRI-4_T7_D11; ARBRI-8_T7_C03 |
| ARBRI-C144 | ARBRI-4_T7_E03; ARBRI-5_T7_H01; ARBRI-7_T7_D08; ARBRI-10_T7_F07 |
| ARBRI-C145 | ARBRI-4_T7_E09; ARBRI-9_T7_D01 |
| ARBRI-C146 | ARBRI-4_T7_F02; ARBRI-6_T7_F10; ARBRI-7_T7_D11 |
| ARBRI-C147 | ARBRI-4_T7_F08; ARBRI-9_T7_G10; ARBRI-6_T7_C06; ARBRI-6_T7_E06; ARBRI-9_T7_H12 |
| ARBRI-C151 | ARBRI-4_T7_G06; ARBRI-9_T7_E01 |
| ARBRI-C152 | ARBRI-4_T7_G07; ARBRI-8_T7_G08 |
| ARBRI-C153 | ARBRI-4_T7_G08; ARBRI-8_T7_F03 |
| ARBRI-C155 | ARBRI-5_T7_A06; ARBRI-9_T7_A05 |
| ARBRI-C156 | ARBRI-5_T7_B11; ARBRI-8_T7_A11 |
| ARBRI-C157 | ARBRI-5_T7_C02; ARBRI-5_T7_F10 |
| ARBRI-C159 | ARBRI-5_T7_C12; ARBRI-9_T7_C11 |
| ARBRI-C161 | ARBRI-5_T7_D06; ARBRI-7_T7_B06; ARBRI-9_T7_C08; ARBRI-9_T7_B06 |
| ARBRI-C164 | ARBRI-5_T7_F08; ARBRI-8_T7_G09 |
| ARBRI-C166 | ARBRI-5_T7_G02; ARBRI-10_T7_B06 |
| ARBRI-C167 | ARBRI-5_T7_G03; ARBRI-6_T7_A06 |
| ARBRI-C168 | ARBRI-5_T7_H03; ARBRI-10_T7_G12 |
| ARBRI-C169 | ARBRI-6_T7_A03; ARBRI-6_T7_G12 |
| ARBRI-C171 | ARBRI-6_T7_C05; ARBRI-9_T7_C05 |
| ARBRI-C173 | ARBRI-6_T7_C09; ARBRI-10_T7_C05 |
| ARBRI-C174 | ARBRI-6_T7_H09; ARBRI-6_T7_E04; ARBRI-7_T7_H02 |
| ARBRI-C177 | ARBRI-7_T7_A06; ARBRI-8_T7_D10 |
| ARBRI-C179 | ARBRI-7_T7_C09; ARBRI-9_T7_B01 |
| ARBRI-C181 | ARBRI-7_T7_E01; ARBRI-9_T7_G03 |
| ARBRI-C183 | ARBRI-7_T7_E08; ARBRI-7_T7_H05; ARBRI-10_T7_C12 |
| ARBRI-C184 | ARBRI-7_T7_E10; ARBRI-9_T7_C06 |
| ARBRI-C185 | ARBRI-7_T7_F02; ARBRI-10_T7_F02 |
| ARBRI-C186 | ARBRI-7_T7_H09; ARBRI-8_T7_G03 |
| ARBRI-C188 | ARBRI-10_T7_A03; ARBRI-8_T7_C12 |
| ARBRI-C189 | ARBRI-8_T7_D11; ARBRI-8_T7_H07 |
| ARBRI-C190 | ARBRI-8_T7_E04; ARBRI-10_T7_G09 |
| ARBRI-C191 | ARBRI-8_T7_E12; ARBRI-10_T7_G10 |
